# Capturing complex tumour biology *in vitro*: histological and molecular characterisation of precision cut slices

**DOI:** 10.1038/srep17187

**Published:** 2015-12-09

**Authors:** Emma J. Davies, Meng Dong, Matthias Gutekunst, Katja Närhi, Hanneke J. A. A. van Zoggel, Sami Blom, Ashwini Nagaraj, Tauno Metsalu, Eva Oswald, Sigrun Erkens-Schulze, Juan A. Delgado San Martin, Riku Turkki, Stephen R. Wedge, Taija M. af Hällström, Julia Schueler, Wytske M. van Weerden, Emmy W. Verschuren, Simon T. Barry, Heiko van der Kuip, John A. Hickman

**Affiliations:** 1 Oncology iMed, Bioscience, AstraZeneca, Alderley Park, Macclesfield, SK10 4TG, United Kingdom; 2 Dr Margarete Fischer-Bosch Institute of Clinical Pharmacology and University of Tuebingen, Auerbachstrasse 112, 70376 Stuttgart, Germany; 3 FIMM, University of Helsinki; Helsinki, Finland; 4 Department of Urology, Erasmus MC Rotterdam, PO Box 2040, 3000 CA Rotterdam, The Netherlands; 5 Institute of Computer Science, University of Tartu, J. Liivi 2, 50409, Tartu, Estonia; 6 Oncotest GmbH, Am Flughafen 12-14, 79108 Freiburg, Germany; 7 Institut de Recherches Servier c/o 126 boulevard Pereire, 75017 Paris, France

## Abstract

Precision-cut slices of *in vivo* tumours permit interrogation *in vitro* of heterogeneous cells from solid tumours together with their native microenvironment. They offer a low throughput but high content *in vitro* experimental platform. Using mouse models as surrogates for three common human solid tumours, we describe a standardised workflow for systematic comparison of tumour slice cultivation methods and a tissue microarray-based method to archive them. Cultivated slices were compared to their *in vivo* source tissue using immunohistochemical and transcriptional biomarkers, particularly of cellular stress. Mechanical slicing induced minimal stress. Cultivation of tumour slices required organotypic support materials and atmospheric oxygen for maintenance of integrity and was associated with significant temporal and loco-regional changes in protein expression, for example HIF-1α. We recommend adherence to the robust workflow described, with recognition of temporal-spatial changes in protein expression before interrogation of tumour slices by pharmacological or other means.

Cancers have been appropriately described as pathological organs^1^,^2^, where the complex interplay between a genotypically and phenotypically heterogeneous tumour and host cells in the stroma plays a key role in tumour development, progression and response to therapy^3^,^4^. Recapitulation of a complex and heterogeneous *in vivo* tissue or pathology by creating appropriate models *in vitro* is a challenging enterprise and studies of cancer cell biology and pharmacology demand that reductionist models attempt to capture, with optimal fidelity, key characteristics of a tumour and its supporting stroma. However, highly reductionist models, especially established cell lines growing in two dimensions on plastic^5^, neither capture three dimensional tumour architecture nor many of the important signalling dynamics of the interactions between a tumour cell and its microenvironment. These signals play important roles in determining key aspects of tumour biology and pharmacology such as cell survival and sensitivity to drugs. Attempts to better represent the complexities of tumour biology, particularly the important architectural features of growth, survival and motility in three dimensions, as well as to represent the gradients of nutrients and oxygen in a solid tumour, are being addressed through investigations of simple or complex tumour three dimensional spheroid models, mostly generated from established cell lines. These spheroid models sometimes have added elements of stroma, such as fibroblasts or endothelial cells^6^,^7^. Recently, by using carefully selected growth media, the generation of tissue and tumour organoids from normal or tumour stem cells *in vitro* has opened up new opportunities for the study of heterogeneous tissue and tumour architecture^8^. Nevertheless, the complexity observed *in vivo* is only partially reconstructed *in vitro* by laboratory manipulation of established cell lines or pluripotent stem cells and components of the stroma are often absent.

A potentially more complete *in vitro* representation of tumour architecture, heterogeneity and the complexity of a tumour *in situ* is provided by precision-cut slices (also known as organotypic cultures). Such 200–300 micron precision-cut slices capture both the three-dimensional architecture and full heterogeneity of the tumour of interest, in addition to potentially retaining important native cellular elements such as immune, vascular and mesenchymal cells^6^. A growing number of publications are using tumour tissue slice culture as a method to address sensitivities to anti-tumour drugs, in the context of precision medicine^9^,^10^,^11^,^12^,^13^. However, there is no standardised approach to cultivating tumour slices, and little is known about the impact of slicing on tissue integrity and function. We wished to determine whether freshly prepared, precision cut slices are capable of capturing and then maintaining essential features of the heterogeneous architecture and biochemistry of an *in vivo* tumour. Although the study has drug target validation and drug discovery in view (www.predect.eu), it is relevant to tumour biology in general, where it is increasingly clear that biochemical circuitry is influenced by tumour architecture and the microenvironment. Towards the goal of characterising precision-cut slices, we first asked what were the biochemical consequences, measured by both selected transcript profiling and immunohistochemistry, of the transport after surgical removal of the *in situ* tumour, the subsequent manipulations necessary to provide precision-cut slices and then of the incubation of the slices themselves under various conditions *in vitro* with time. Such fundamental questions need to be answered before a slice can be interrogated as an appropriate surrogate for an *in situ* tumour; the potential trauma of removing, transporting and then slicing the tumour in addition to its subsequent incubation *in vitro* may “rewire” the biochemical circuitry of both the tumour and stromal cells. A major concern was the possible imposition of tissue stress responses on the biochemistry of slices and we asked, if so, whether this stress could be ameliorated.

We here address the technical issues regarding preparation and maintenance of precision-cut slices, using readily available tumour tissue from mouse models of human breast, prostate and lung cancers derived from both cell lines (CDX) and patient-derived human tumours (PDX) as xenografts, as well as a genetically engineered mouse model (GEMM) of non-small cell lung cancer. These models were necessary surrogates for intensive technical studies that could not be readily performed with fresh human tumours, given their limited availability, intra-tumoural heterogeneity and reproducibility. We describe a workflow for slice preparation, cultivation and analysis that is amenable to bespoke optimisation. We propose this as a prerequisite baseline prior to planning any model-pertinent functional studies, such as drug perturbation. Additionally, to permit continuing interrogation of protein expression in slices by immunohistochemistry, we describe a novel method for preparing tumour slices as tissue microarrays (TMAs), permitting systematic archiving of large numbers of slice samples.

## Results

### Atmospheric oxygen levels and filter supports are required for retention of tissue morphology in slices

**Figure 1:**
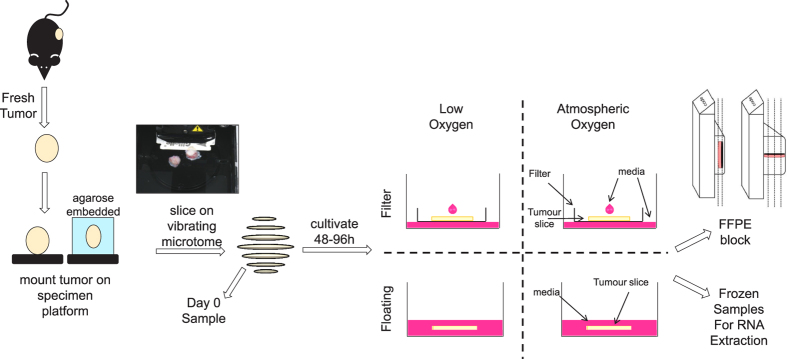
Tumour tissue slice culture workflow. Fresh tumours were harvested from the mice and mounted on a tumour specimen platform, either directly or mounted in agarose. The Leica VT1200S vibrating microtome was used to cut slices of 200–300 μm in thickness. Some sliced samples were fixed and frozen at day 0 prior to cultivation and the remainder were cultivated. Experiments were performed to compare the impact of filter supports and oxygen levels on tissue viability, which was estimated using either histological, immunohistochemical or RNA transcript-based biomarkers. Slices were sectioned both horizontally and vertically for analysis of morphology and of protein expression by immunohistochemistry.

**Figure 2:**
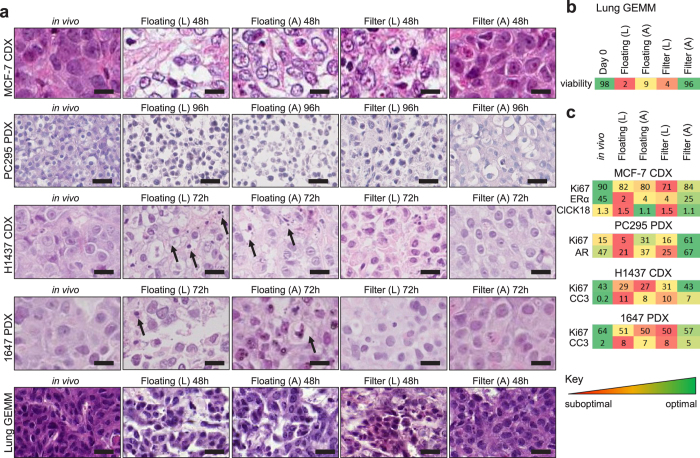
Histopathology of cultivated tumour slices. (**a**) High power images of H&E stained tumour tissue slice sections, representing each tumour model cultivated under different conditions, compared to a stained section of the original *in vivo* tumour (CDX = cell line-derived xenograft; PDX  = patient-derived tumour; GEMM  = genetically engineered mouse model). Rows H1437 CDX and 1647 PDX: arrows indicate condensed nuclei (apoptosis) and vaculated regions. Representative images from at least three experiments are shown. Scale bars represent 25 μm. (**b**) Heatmap of the percentage of viable tissue in NSCLC GEMM tumour slices determined by the algorithm described in the Methods. **(c**) Heatmaps representing the percentage of positive cells expressing viability markers by immunohistochemistry (IHC) of slices cultured under different conditions (numbers in heatmap boxes represent the percentage of positive cells for each biomarker). Key to conditions: A – atmospheric oxygen, L – low oxygen. Ki67, indicating proliferation; ClCK18, cleaved cytokeratin 18 indicating apoptosis; ERα, estrogen receptor-alpha; AR, androgen receptor; CC3, cleaved caspase 3. The values reflect means from three independent experiments.

We first compared previously published methods of slice cultivation to determine which conditions achieved maximal retention of tissue morphology and integrity. For this, tumour slices from breast, prostate and lung cancer *in vivo* models were cultivated for 48–96 h, either immersed in media (floating), or on a Millicell® filter support at an air-liquid interface (filter). Tumour slices were cultivated in either low oxygen conditions (L, <5% O_2_) or in atmospheric oxygen (A, ~20% O_2_) (Fig. 1). Qualitative evaluation of H&E stained horizontal sections of tumour slices from all tumour types showed that cultivating slices on a filter support maintained a tissue morphology which was most similar to *in vivo* tissue morphology (Fig. 2a). In contrast, cultivation of floating tumour slices induced condensed apoptotic nuclei and vacuolated structures (Fig. 2a, arrows). Oxygen levels appeared to have little impact on the overall morphology of the lung H1437 CDX and 1647 PDX tumour slices. Interestingly, however, low oxygen impacted negatively on the morphology of MCF-7 CDX, PC295 PDX and lung GEMM tumour slices even when cultivated on filters (Fig. 2a). This was confirmed using a tissue segmentation algorithm, developed to quantify the level of viable and necrotic tissue present in H&E stained sections from lung GEMM tumour slices. 96% of the GEMM tissue was found to be viable when cultivated in atmospheric oxygen on a filter whereas viability was below 10% in all other conditions (Fig. 2b). The need for high oxygen levels as well as organotypic filter support for maintaining tissue integrity in MCF-7 CDX tumours was confirmed by the finding that cell-cell contacts were lost when slices were cultivated at low oxygen tension and/or as floating cultures, as revealed by E-cadherin staining (Supplementary Fig. 1a). Analogous results were found regarding hormone receptor expression in breast and prostate tumour slices. Expression of the oestrogen receptor (ER) and androgen receptor (AR) was sustained only in the presence of atmospheric oxygen together with filter supports (Fig. 2c, Supplementary Fig. 1b,e). Finally, IHC staining for proliferation and apoptosis indicates that both atmospheric oxygen and organotypic filter supports are important to retain viability (Fig. 2c, Supplementary Fig. 1b–e).

Using histopathological methods we conclude that the viability of tissues, derived from different tumour models (CDX, PDX, GEMM) and pathologies (breast, lung, prostate), is best maintained when slices are cultivated on organotypic filter supports with atmospheric oxygen. Preliminary experiments with two primary human ER positive (ER^+^) breast tumours showed that the importance of using a filter support for primary tissue viability was variable. Slices from one tumour required a filter support for retention of ER^+^ cells. However, ER^+^ cells were retained in slices from a second tumour cultivated both with and without filter supports (Supplementary Fig. 1g).

The reproducibility of ER expression in tissue slices of four ER positive breast cancer MCF-7 CDX tumours cultivated on organotypic filter inserts with atmospheric oxygen is illustrated in Supplementary Fig. 1f. Expression of the ER was assessed immediately after slice preparation (0 h), or after 48 h of cultivation (Supplementary Fig. 1f). ER levels observed immediately after slicing were maintained similarly to those observed *in vivo*. After cultivation of MCF-7 tumour slices for 48 h, ER levels in each individual slice remained similar, with little variability between slices. Similar results were obtained using slices from the PC295 prostate PDX (data not shown) supporting good inter-experimental reproducibility.

### Cultivation of tumour slices induces changes in key stress pathways

To complement the histological analysis of slices and to have a wider view of the impact of slice cultivation on tissue viability, we analysed changes in expression of genes thought to be biomarkers related to cellular stress in tumour slices using Fluidigm microfluidic dynamic qPCR arrays (48.48 and 96.96 chip formats), in combination with 134 TaqMan® assays. The choice of transcripts was either based on a known role of the respective proteins in cellular stress pathways (<15% of the transcripts) or according to the following criteria (>85%): (i) they are typical targets of stress-activated transcription factors (such as p53 or NFkB), (ii) they are differentially regulated upon inhibitors/siRNA treatment specifically blocking certain stress pathways (such as the p38/JNK pathway) or (iii) the promoter regions of their genes have been described to contain stress response elements (such as endoplasmic reticulum (er) stress response elements). A full list of transcripts and their functional classification can be found in Supplementary Table 1. We hypothesised that the conditions for preparation of slices (the tumour transport and cutting) and their subsequent incubation were both likely to induce stress. Tumour slices were analysed at either 48 h or 72 h (H1437 CDX and 1647 PDX), and an additional sample that had been sliced but not cultivated (day 0) was analysed to assess the immediate impact of transportation and mechanical slicing on biomarker expression following tumour excision (Fig. 1).

**Figure 3:**
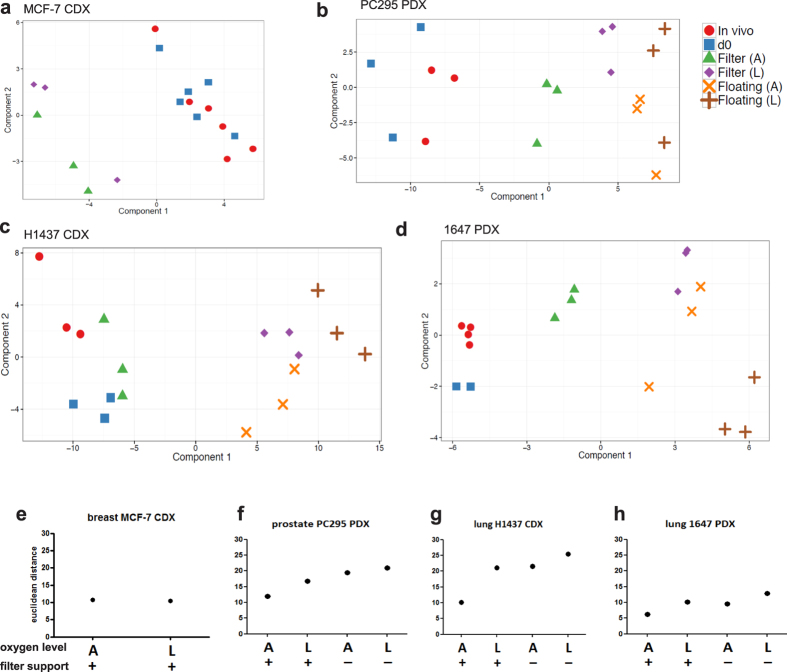
Evaluation, by Principal Component Analysis (PCA), of the changes in stress biomarkers observed under different conditions of slice culture. (**a**–**d**) PCA plots of the expression of each of the 134 stress genes quantified under different conditions of slice culture. (**e**–**h**) Scatterplots of the Euclidean distance of stress gene biomarker expression profiles of tumour slices compared to the expression profiles of the corresponding *in vivo* tumours. Key: A- atmospheric oxygen; L-low oxygen; +  = cultivation with a filter support; − = cultivation without a filter support. Results are from three independent experiments.

**Figure 4:**
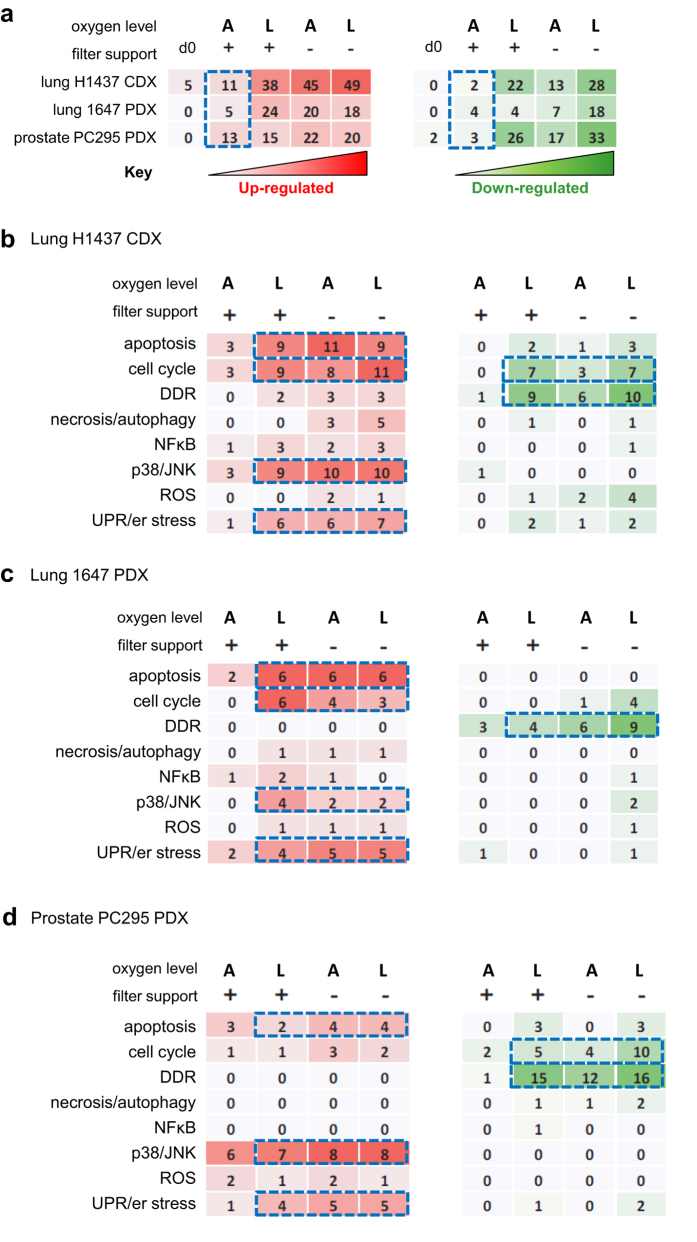
Pathway changes induced by tumour slice culture. (**a**) Table showing the numbers of differentially expressed genes (DEGs) in prostate and lung models cultivated under different conditions. (**b**–**d**) Tables showing the numbers of DEGs under each cultivation condition and their associated functions for both of the lung tumour models and the prostate tumour model. Key: A- atmospheric oxygen, L-low oxygen, + cultivated with a filter support, –cultivated without a filter support, red  = increases in expression; green = decreases in expression. Results are from 3 independent experiments.

To compare the overall impact of changes in stress related biomarkers for each cultivation condition, we used Principal Component Analysis (PCA) and Euclidian distance scores (Fig. 3). Importantly, only limited stress biomarker transcript expression changes were detected in the tissue slice prior to cultivation, i.e. directly after slicing (day 0; Fig. 3a–d, Fig. 4a). In accordance with the histological observations (Fig. 2), the data showed that filter-supported cultures in atmospheric oxygen clustered closest to *in vivo* and day 0 samples except for MCF-7 (Fig. 3a–d). This was confirmed by Euclidean distance scores that represent the relationship between cultivated slice samples and the *in vivo* samples (Fig. 3e–h) and by the absolute number of significantly changed transcripts (Fig. 4a). The fold-change of individual biomarker transcripts is represented in Supplementary Table 2.

Although changes in biomarkers were generally consistent between the different models, we identified changes specific to individual models that would require consideration when using slices for functional studies. For example, in our hands, MCF-7 CDX tissue slices cultivated floating without filter supports were observed to undergo a substantial loss of tissue architecture and cell viability (Fig. 2a). As a consequence, insufficient RNA of quality was able to be extracted for biomarker analysis. Unlike the lung and prostate models, MCF-7 CDX tissue slices, cultivated on filter supports either in atmospheric or low oxygen, clustered together in the PCA plot and Euclidean distance scores showed no difference between the two conditions of oxygenation (Fig. 3a,e, Supplementary Fig. 2a,b). This is in contrast to the histological findings where tissue integrity and cell-cell contacts were lost in slices cultivated in low oxygen on filters (Fig. 2, Supplementary Fig. 1a,b). Detailed examination of MCF-7 CDX tissue slices cultivated on filters in low oxygen revealed that the majority of cells that experienced a lack of oxygen had poor viability (Supplementary Fig. 2c). This highlights the need for a suite of biomarker approaches, both histological and pathway-based, to drive baseline characterisation and refinement of slice cultivation protocols for individual tumour types.

Classification of stress-related transcripts according to their involvement in cellular processes (Supplementary Table 1) revealed that the majority of the transcripts down-regulated during slice cultivation, under conditions classed as suboptimal (low oxygen and/or without filter support), are involved in DNA damage response (DDR) pathways. Importantly, this pattern of change was seen for all model systems under suboptimal conditions (Fig. 4b–d). Although important changes in post-transcriptional regulation of the DDR during incubation under suboptimal conditions could be investigated further, these suboptimal conditions are not recommended for detailed interrogation of slices. Up-regulation of transcripts was predominantly observed for apoptosis-, er stress- and p38/JNK-related genes, and again was observed across all the models. In the two lung models, cell cycle-related transcripts were found to be up-regulated (Fig. 4b,c, Supplementary Table 2), whereas in prostate PC295 PDX and breast MCF-7 CDX slices, cell cycle related transcripts were generally down-regulated. To determine if there were transcriptional changes common to all models, Venn diagrams were generated, comparing transcriptional changes occurring under both optimal (air, filter support) or suboptimal conditions (Supplementary Fig. 3). Under optimal conditions, comparing the changes in gene expression observed in three different xenografted cancers, only one gene, ATF3, was up-regulated in common. ATF3 is implicated in the unfolded protein response (UPR) and the endoplasmic reticulum (er) stress response (Supplementary Fig. 3a). A number of statistically significantly altered gene transcripts related to apoptosis, UPR/er stress and DDR were observed according to the particular model and conditions of incubation (Supplementary Fig. 3b). Under suboptimal cultivation conditions (no filter support or low oxygen with filter support), the down-regulation of BRCA1 and BLM (DDR) and up-regulation of HERPUD1 (see also Supplementary Table 1) was common to slices from all three tumour xenograft models (Supplementary Fig. 3c). These three changes, observed in common, could be investigated further as potential biomarkers of suboptimal slice cultivation conditions when analysis of the number of different models is increased. Under suboptimal conditions there were other changes which were particular to slices derived from each pathology (Supplementary Fig. 3d).

### Cultivation at an air-liquid interface, with a filter support, induces loco-regional changes in biomarkers

**Figure 5:**
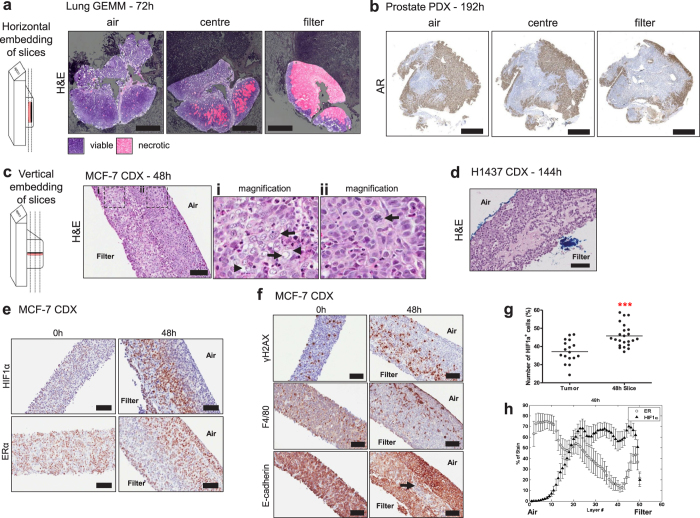
Loco-regional changes in IHC biomarkers in tumour slices cultivated under optimal conditions (filter support, atmospheric oxygen). (**a**) Lung GEMM tumour slices, horizontally embedded, were found to contain large areas of necrosis at the filter interface of the slice. Scale bars represent 1000 μm. (**b**) AR staining revealed that AR positive cells were in abundance at the air interface in prostate PDX tumour slices and were greatly reduced at the filter interface. Scale bars represent 1250 μm. (**c**) H&E stained sections of vertically embedded MCF-7 CDX tumour tissue slices. Images show differences in tissue morphology dependent on their proximity to the air interface of the slice. (i) Arrows: areas of necrosis; arrowheads, vacuolated regions (ii) Arrow: mitotic figure. (**d**) H&E stained section of vertically embedded H1437 CDX tumour slice, showing similar morphology to that observed in MCF-7 tumour slices. (**e**) Immunohistochemical staining of HIF1α in MCF-7 tumour slices showed an accumulation of nuclear protein at the filter interface of the slice, and immunohistochemical staining of ER, which showed a reduction in staining at the filter interface. Scale bars represent 100 μm. (**f**) Immunohistochemical staining for γH2AX indicated an abundance of foci at the filter interface. F4/80 immunohistochemical staining showed that there was a propensity for F4/80 positive macrophages to be found closest to the air interface. Membranous E-cadherin was present at both the filter and air interfaces. Scale bars represent 100 μm. (**g**) The variability of HIF1α expression observed in MCF-7 CDX tumours *in vivo* compared with that in MCF-7 slices after 48 h (n = 17 tumours, 23 slices, ***p value < 0.0001) (**h**) The spatial distribution of HIF1α and ER across the thickness of the slice was quantified using a segmentation algorithm (see Methods). Each slice image was divided into 50 layers, 0 representing the layer closest to the air interface and 50 closest to the filter interface. Error bars represent standard deviation, n = 7 cultivated slices from three tumours.

A tissue slice is a complex structure of finite depth and in order to evaluate loco-regional changes across the slice, formalin-fixed tumour slices were embedded in paraffin wax and either sectioned horizontally (Fig. 5a,b) or vertically (Fig. 5c–f). H&E and immunohistochemical staining revealed changes in histology and protein expression patterns, denoting differences in viable and necrotic areas, when comparing the filter interface to the air interface of the tissue (Fig. 5). Slices of the lung GEMM tumour were found to contain larger areas of necrosis at the filter interface of the slice, shown by H&E staining (Fig. 5a). In slices of the PDX prostate tumour, although necrotic areas were not observed, AR positive cells were only in abundance at the air interface (Fig. 5b). In a vertically sectioned slice of the MCF-7 CDX, the slice surface adjacent to filter showed vacuolation and evidence of apoptosis (Fig. 5c(i), arrows) whereas a thin layer of cells at the air interface contained tightly packed cells with evidence of mitotic figures (Fig. 5c[ii], arrow). Taken together, within two to four days of culture on organotypic filter supports, all models showed a proliferation/viability gradient with loco-regional changes in morphology (eg. Fig. 5d) and biomarker expression.

The gradients of biomarker expression observed were particularly associated with the morphological changes observed in the MCF-7 CDX tissue slices in close proximity to the filter support and were coincident with accumulation of nuclear hypoxia inducible factor α (HIF1α) (Fig. 5e) and γH2AX foci, a marker of double strand breaks in DNA, indicating that cells in this slice compartment were hypoxic and stressed (Fig. 5f). The hormone receptor ER drives proliferation of MCF-7 cells^14^ and the reduction of ER positive staining in the filter region of the slice (Fig. 5e), was associated with HIF1α expression. There was variable expression of HIF1α in the MCF-7 xenografts *in vivo* and this variability was captured in the slices where, on cultivation under optimal conditions, hypoxia increased significantly (Fig. 5g). The inverse relationship between HIF1α and ER expression has been described previously^15^. After 48 h of incubation, quantification of HIF1α and ER in a spatial manner across the slice thickness was carried out using bespoke software (see Methods), quantitatively confirming an accumulation of HIF1α at the filter region of the slice and a coincident depletion in ER levels (Fig. 5h). After 48 h of incubation, slices measured 357 + 55.2  μm (n = 7) estimated by using this software. MCF-7 slices were also cut at thicknesses between 175 μm and 250 μm and the expression of HIF1α assessed (Supplementary Fig. 4). The expression of HIF1α was not discernably different according to slice thickness.

As an assessment of mouse host infiltrate, IHC was performed with an antibody specific to mouse macrophages (F4/80 positive cells). This revealed that host stroma was retained in MCF-7 tumour slices, with macrophages detectable largely at the oxygenated region of the slice close to the air interface after 48 h (Fig. 5f). The distribution of macrophages may be driven by migration, as indicated by pathway analysis of gene expression data (data not shown), and/or their death in the developing hypoxic areas of the slice. In contrast, membranous E-cadherin was retained at both the filter and air interface, but was noticeably reduced in the centre of the slice (Fig. 5f).

**Figure 6:**
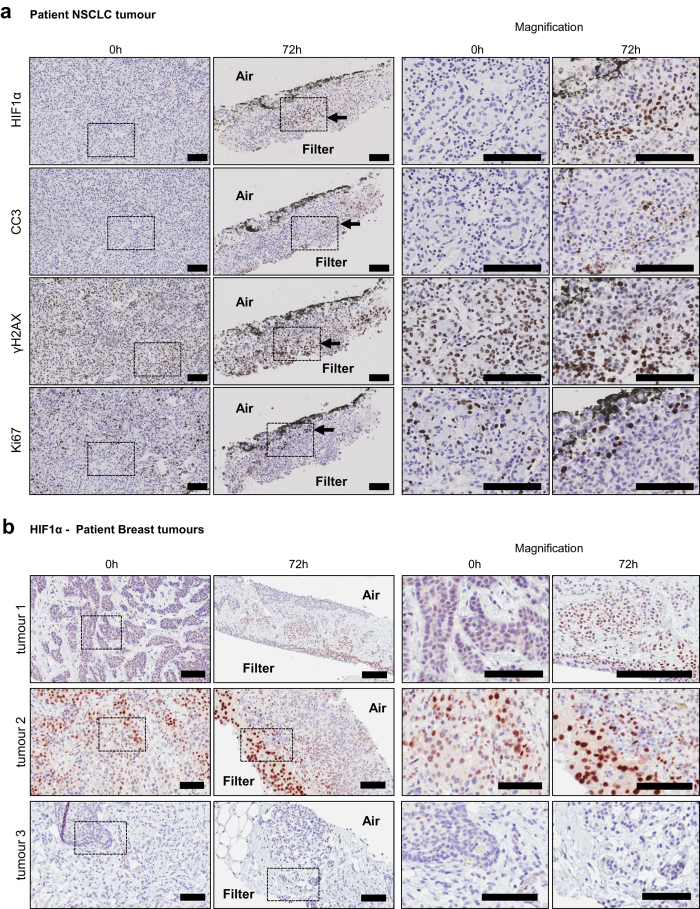
Observations made in murine CDX and PDX tumours were confirmed in patient tumour samples. (**a**) A NSCLC tumour from a patient was obtained, sliced and cultivated on filters for 72 h following the standard workflow. After this time, induction of HIF1α was observed at the filter interface of the slice. This was coincident with increased cleaved caspase 3 (CC3) and γH2AX staining. Conversely, Ki67 positive cells were found at the air interface. (**b**) The induction of HIF1α at the filter interface was also observed in breast tumour slices prepared from samples of patient tumours. Slices from tumours 1 and 2 show a clear accumulation of HIF1α at the filter interface, while slices from tumour 3 did not develop an accumulation of HIF1α after 72 h. Scale bars represent 100 μm.

The loco-regional changes in biomarkers that developed as slices from human xenograft tumours (CDX and PDX) and a GEMM were incubated with a filter support in atmospheric oxygen, were also observed when the work-flow was applied to samples of human patient tumours (Fig. 6). In tissue slices from primary NSCLC, similar differences in the expression of the HIF1α and γH2AX proteins were observed when comparing air and filter interfaces (Fig. 6a). In primary human breast tumours HIF1α was observed in 3 out of 4 tumours cultivated as slices with varying degrees of accumulation at the filter interface (Fig. 6b).

### The type of slice support system impacts upon stress biomarker expression

**Figure 7:**
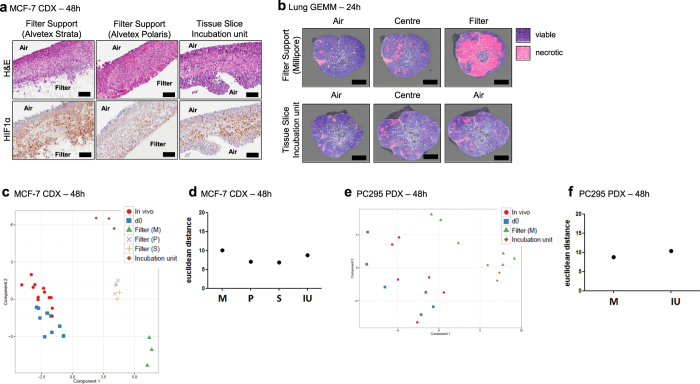
Comparison of slice support materials and slice incubation cultivation systems. (**a**) H&E stained and HIF1α immuno stained sections of MCF-7 CDX tumour slices cultivated using an adherent filter support with a 5 μm pore size (Alvetex Strata), a 0.4 μm pore size (Alvetex Polaris) and using a tissue slice incubation unit supported by titanium grids, which were periodically rotated into the media and air (see Methods). (**b**) H&E stained sections of Lung GEMM tumour slices annotated for regions of necrosis in slices cultivated using Millipore filters and a tissue slice incubation unit. Pink shaded areas indicate necrosis. (**c**–**f**) PCA plot of the stress gene biomarker expression profiles and corresponding euclidean distance scatterplots of MCF-7 CDX and PC-295 PDX tumour slices cultivated using different support methods. Scale bars represent 100 μm. Key: M  = Millipore Filter, P = Alvetex Polaris Filter, S = Alvetex Strata Filter, IU = Tissue slice incubation unit. Results are from three independent experiments.

Having established that filter-supported cultivation was optimal, although associated with loco-regional changes in biomarker expression, we next investigated whether alternative support materials and incubation methodology influenced the loco-regional changes. Changes in the type of the slice support material and methods of incubation indeed influenced the slice architecture and stress biomarker profile. Two types of Alvetex adherent filter supports and a tissue slice incubation unit that permits intermittent immersion of the slice into the media, were tested (Fig. 7). Intermittent immersion is a technique that has the potential to prevent establishment of a filter-air interface gradient, permitting oxygen access to both surfaces of the slice (see Methods). Consistent with the observation using Millipore filters, tumour slices cultivated statically using both types of Alvetex support accumulated HIF1α at the media interface (Fig. 7a). Using the tissue slice incubation unit, MCF-7 tissue slices were found to show expression of HIF1α only in the centre of the slice with both air sides staining negatively, indicative of an oxygen gradient from the two surfaces of the slice to the centre (Fig. 7a). When slices were cultivated using the incubation unit or with Alvetex or Millipore supports, PCA plots and Euclidean distance scores of stress biomarker transcript expression showed there were no differences between Millipore supports and the incubation unit for both breast CDX and prostate PDX slices (Fig. 7c,d,f). For breast CDX slices fewer changes in stress biomarker transcript expression were observed with the Alvetex supports (Fig. 7d). Importantly, cultivation of tissue slices from GEMM lung tumours using the incubation unit noticeably reduced the onset of necrosis observed at the filter side of Millipore supports (Fig. 7b). These data support the use of a range of cultivation platforms that can be assessed for each novel model to be investigated, offering the potential for modification of the oxygen and media gradients as appropriate.

### Archiving of tumour slices

**Figure 8:**
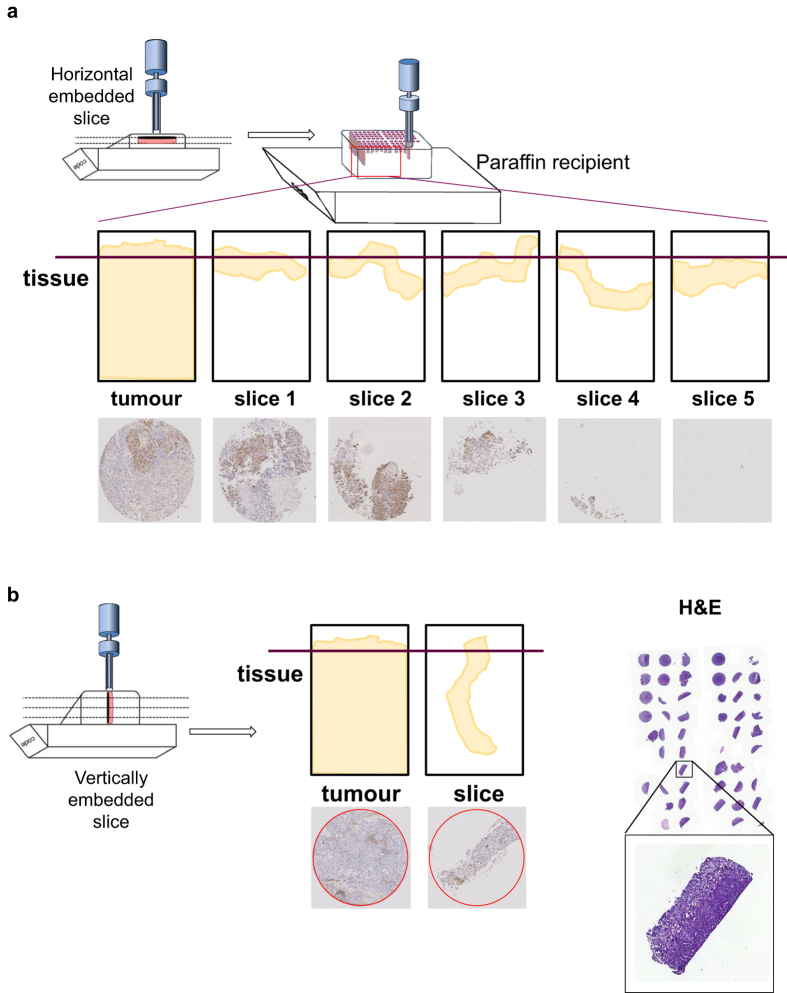
Tissue Micro Array (TMA) workflow for the archiving of tissue slice material. (**a**) Schematic diagram of the generation of TMAs from horizontally embedded tumour slices, and the resultant incomplete tissue sections from these blocks. (**b**) Schematic diagram of the generation of TMAs from vertically embedded tumour slices, and the improvement in achieving consistent and complete tissue sections from these blocks.

To permit further interrogation of tumour tissue slices by IHC and other FFPE methods, we tailored a method to punch tissue slice FFPE blocks and produce a tissue microarray (TMA). Using standard TMA punching to generate a tissue slice TMA from horizontally embedded tumour slices was challenging, as only small amounts of tissue material were present at the sectioning face, as shown in Fig. 8a. Vertically embedded tissue slices (see materials and methods) permitted robust and reproducible tissue slice punches (Fig. 8b).

## Discussion

Tissue slice culture has the potential to permit the interrogation of complex, heterogeneous material from either human tumours or appropriate models whilst the tumour microenvironment is maintained. There are diverse publications suggesting that tumour tissue slice culture can be utilised productively using different cultivation protocols (reviewed in^6^). Studies so far have predominantly been performed using patient tumour samples. These reports are limited by their minimal biochemical characterisation of the impact of either slice preparation or cultivation conditions. Furthermore, a systematic comparison between different cultivation methods is lacking. This is mainly due to the difficulty in defining adequate controls using heterogeneous patient tumour tissues and the impossibility of showing reproducibility of results from *ex vivo* slices obtained from one unique human tumour. Across four independent laboratories, we have developed a unified workflow using surrogate tumours from GEMM and xenograft models, a workflow which can also be applied to primary human tumours and may be adapted to other cancer pathologies. The consistent availability of this surrogate source of accessible and homogenous tumour material allowed us to systematically compare the *in vivo* tumour with tissue slices immediately after preparation and upon cultivation under various conditions. The use of such models to prepare slices is anyway pertinent to fulfilling the needs of investigators when multiple, repeated studies of tumour biochemistry or pharmacology are to be performed or when intensive efforts in industry are required, such as for target validation. We established a biomarker platform to determine how preparation and/or cultivation might influence the activity of stress related pathways in tumour slices. In our hands, a single palpable murine tumour (minimum of 400mm^3^) was able to provide around 20 slices, permitting multiple investigations *ex vivo* of a single complex *in vivo* tumour, with the potential to reduce the use of animals in some studies. Importantly, both vertically and horizontally embedded slices were interrogated by microscopy and immunohistochemistry, permitting an analysis of the status of the interior of the slice and not only of its surface, as reported in other studies.

Importantly, we found for all three tumour types that tumour transportation and slicing had little impact on stress gene expression, but that the act of cultivation itself induced significant changes in gene and protein expression. The comparison of differently cultivated tissue slices revealed that the key parameters governing tissue viability are organotypic filter supports and oxygen levels. When cultivated as floating slices in low oxygen there was a rapid and significant alteration of a number of stress pathways, accompanied by a loss of tissue integrity. This was ameliorated when slices were cultivated on organotypic filter inserts and, somewhat surprisingly, in atmospheric oxygen. Hypoxia is a hallmark of most solid tumours with oxygen levels of <2% (mild hypoxia) and <0.1% (severe hypoxia)^16^. Tissue normoxia is also far away from atmospheric oxygen with ~5% in the brain and ~6% in the lung^17^. Due to the loss of the intact circulatory network in a slice, the availability of oxygen in tissue slices is strictly limited to gas diffusion. This produces an oxygen gradient throughout the tissue slice analogous to that observed around vessels *in vivo*^18^. We presume that atmospheric concentrations of oxygen produce hyperoxic conditions at the air side but allow a supply of just sufficient oxygen to deeper cell layers, distant from the air side. This is supported by our observation that in 3–5% oxygen only a very thin layer of cells at the air side survived. Importantly, histological analysis of organotypic filter-supported tissue slices, from either the murine models or human cancer tissue showed that a local microenvironment was established, orientated between the air and filter interfaces. The development of loco-regional heterogeneity indicates that prudence is required when interpreting the data from studies such as pharmacological perturbation of a slice or explants^9^. However, gradients of oxygen tension are a common feature of solid tumours^16^ so that these conditions offer the potential to recapitulate an important aspect of *in vivo* tumour biology *in vitro*.

The kinetics of the onset of hypoxia in incubated slices was variable amongst the models and pathologies studied here, with slices from the MCF-7 CDX showing expression of HIF1α after only 24 h. The diffusion of oxygen and its utilisation will be controlled by several variables in a slice lacking a functional vasculature. Perhaps surprisingly, slice thickness was not a major determinant (Supplementary Fig. 4). Other potential factors include matrix stiffness, cellularity and the metabolic state of both carcinoma and stromal cells, including their proliferative status. It would be necessary to compare the stromal status (density of fibroblasts, vascular cells) and cellularity of a large series of tumours, preferably of the same pathology, together with their metabolic status before conclusions about determinants of the onset of hypoxia could be firmly determined. One of us (STB) determined that the stromal status of a variety of xenografted tumours was variable^19^. This provides the basis for a further in depth study of variables controlling the kinetics of the onset of hypoxia.

Our standardised workflow for tumour tissue slice preparation and culture, using filter support in atmospheric oxygen, enables implementation of a robust and routine platform. Importantly, when establishing tissue slice cultures it is essential to generate a baseline biomarker profile of the model of interest, analysing vertical sections of the slice, so that multiple samples from the same tissue of origin or from different pathologies can be compared. From this baseline it should be possible to develop the platform further, integrating developments in microfluidics and perfusion systems in order to optimise oxygen supply and further reduce cultivation-induced cell stress, thus permitting longer-term culture. The platform permits future evaluation of the fidelity of signalling circuitry and the role of the native stroma in a heterogeneous cancer by immunohistochemical methods, using the archived TMAs. This offers a very powerful technique to investigate tumour biology and pharmacology in the context of a tissue, something that is missing from alternative *in vitro* models. Although advances have been made in generating cell lines from human cancers, for example from drug-resistant lung cancers^20^, unlike slices these often require specialised media, risking the selection of cells from a heterogeneous tumour. The use of slices also provides a means of interrogating tumours from PDX and advanced GEM models^21^,^22^ where it is sometimes difficult to derive cell lines for *in vitro* studies, and avoids the use of disaggregation systems. Tissue slice culture can be a valuable addition to the suite of *ex vivo or in vitro* assays that are used currently to study tumour biology, pharmacology and for the validation of tumour biomarkers on primary human tissue.

## Methods

### Tumour material from mice

All animal facilities and handling protocols were approved by the local committees on ethics of animal experiments, as required in each country, and adhered to the European Convention for Protection of Vertebrate Animals used for Experimental Purposes (Directive 2010/63/EU). Experiments carried out with mice at AstraZeneca were compliant with the UK Animals (Scientific Procedures) Act, which is consistent with EU Directive 2010/63/EU and had undergone internal ethical review. Experiments with mice performed at Oncotest were scrutinised by the Committee on the Ethics of Animal Experiments of the regional council (Regierungspräsidium Freiburg, Abt. Landwirtschaft, Ländlicher Raum, Veterinär- und Lebensmittelwesen). In Helsinki, studies with mice followed guidelines from the Finnish National Board of Animal Experimentation, and were approved by the Experimental Animal Committee of the University of Helsinki and the State Provincial Office of Southern Finland (Licence number ESAVI-2010-04855/Ym-23). In Rotterdam, the protocols for the use of mice were approved by the Animal Experiments Committee, DECConsult, an independent committee responsible for accreditation of all animal experimentation under the Dutch Experiments on Animals Act and all protocols were reviewed so to adhere to the European Convention for Protection of Vertebrate Animals used for Experimental Purposes.

### Cell line-derived xenograft (CDX) tumour samples

The CDX MCF-7 was derived by subcutaneous injection of 5 × 10^6^ MCF-7 cells (American Type Culture Collection) per 0.1 ml in 50:50 basal media and Matrigel (BD Biosciences) into the left flank of male SCID mice (SCID/CB17). Animals were implanted with 0.5 mg/21 day 17β oestradiol pellets (Innovative Research of America) one day prior to cell implant.

The CDX NCI-H1437 was derived by subcutaneous injection of 5 × 10^6^ NCI-H1437 cells (American Type Culture Collection) into the flank of 4–6 weeks old NMRI nu/nu mice.

### Patient-derived xenograft (PDX) tumour samples

PDX from lung cancer (LXFA 1647) was derived from a metastasis in the adrenal gland of a 53 year old woman. 4–6 week old female NMRI nu/nu mice under isoflurane anaesthesia receive tumour implants subcutaneously in the flank.

PDX of prostate cancer (PC295) was established as described previously^23^. This PDX was routinely passaged by subcutaneous grafting of small fragments into both shoulders of intact male athymic NMRI nu/nu mice (Taconic) under isoflurane anaesthesia. Mice were supplemented with self-made testosterone-containing silastic implants as described previously^24^.

### GEMM-derived primary NSCLC tumours

The conditional *LSL-Kras*^*G12D/+*^*; Lkb1*^*fl/fl*^ (*Kras;Lkb1*) GEMM was used and maintained on a mixed genetic background. LSL-Kras^*G12D/+*^^25^ mice were purchased from Jackson Laboratories and *Lkb1*^*fl/fl*^^26^ were shared by Ron DePinho (Houston, TX, USA). Mice were anaesthetised with isoflurane, and tumour growth was initiated by intranasal delivery of Adenoviral CMV-Cre (3 × 10^7^ pfu/mouse; Gene Transfer Vector Core) in 60 μL volume, as previously described^27^.

### Tumour harvesting

Xenografted tumours were harvested when the volume was between 0.4–1 cm^3^. Animals were euthanised by cervical dislocation and tumours were excised. A small sample of the tumour was either snap frozen or fixed in 10% neutral buffered formalin immediately after resection. Tumours were placed into ice-cold Oncostore Tumour storage solution (OncoScience AG) or ice cold Leibovitz L15 medium (Lonza) prior to tissue slicing. NSCLC GEMMs were euthanised by cervical dislocation when they showed laboured breathing. Lungs *en bloc* were harvested for tissue slicing or fixation with formalin.

### Primary Patient Tumours

Breast tumour samples were obtained with fully informed consent from Wythenshawe hospital and transferred to AstraZeneca in ice-cold Oncostore tumour storage solution (OncoScience AG) on the day of surgical excision. The use of these samples was approved by AstraZeneca and The Manchester Cancer Research Centre Biobank (http://www.mcrc.manchester.ac.uk/biobank/), which operates under HTA licence number 30004 and NRES ethics number 07/H1003/161+5 in accordance with the approved guidelines. Fresh sterile tumour tissue of NSCLC patients was obtained as surgical waste from Klinik Schillerhöhe, Stuttgart immediately after surgical resection and maintained in organ transportation medium (Eurocollins, Germany) until use. The investigation was approved by the local ethics committee (159/2011BO2) and informed consent was obtained from the patients.

### Tissue slice preparation and cultivation

Tumours were mounted onto the magnetic specimen holder of a Leica VT1200S vibrating blade microtome using cyanoacrylate adhesive. Tumours of small volume were first embedded in low temperature gelling agarose (Thermo) prior to mounting on the specimen holder. Tumour tissue slices were prepared at a thickness of 200 μm (mouse lung), 250 μm (lung and breast) and 300 μm (prostate) using the precision-cut vibrating-blade microtome (Leica VT1200S). Slices of <175 μm were found to be too fragile to manipulate. Slices were visually inspected whilst being cut to ensure the tissue was not compressed or torn, resulting in inconsistent slice thickness. It is of note that 3/21 primary human breast tumours received were unable to be sliced; the dense consistency of these tumours prevented the blade from cutting the tumour. Lung PDX and CDX slices were cultivated in RPMI 1640, GEMM tumour slices were cultivated in F12 (Gibco), MCF-7 CDX slices were cultivated in DMEM (Sigma). Medium was supplemented with glutamine (2 mM; Gibco), penicillin (100 U/ml; Gibco), streptomycin (100 μg/ml; Gibco) and 10% foetal bovine serum (FBS; Gibco). Prostate PDX slices were cultivated in DMEM/F12 medium (Lonza, Belgium) supplemented with 2% FBS (Gibco), 1% insulin-transferrin-selenium (Gibco BRL), 0.01% bovine serum albumin (Boehringer Mannheim), 10 ng/ml epidermal growth factor (Sigma-Aldrich), penicillin/streptomycin antibiotics (100 U/ml penicillin, 100 μg/ml streptomycin; Lonza) plus the following additions: 100 ng/ml fibronectin (Harbor Bio-Products), 20 μg/ml fetuine, 50 ng/ml cholera toxin, 0.1 mM phosphoethanolamine, 0.6 ng/ml triiodothyronine, 500 ng/ml hydrocortisone and 0.1 nM synthetic androgen R1881 (all from Sigma-Aldrich)^28^. Primary breast tumour slices were cultivated in mammary epithelial cell medium supplemented with bovine pituitary extract (0.004 ml/ml), Epidermal Growth Factor (recombinant human, 10 ng/ml) insulin (recombinant human, 5 μg/ml), hydrocortisone (0.5 μg/ml, all from Promocell) and penicillin (100 U/ml; Gibco), streptomycin (100 μg/ml; Gibco). Primary NSCLC tumour slices were cultivated in RPMI 1640 supplemented with glutamine (2 mM; Gibco), penicillin (100 U/ml; Gibco), streptomycin (100 μg/ml; Gibco) and 10% fetal bovine serum (FBS; Gibco).

Cultivation was performed at 37°C and 5% CO_2_ in a humidified atmosphere under low oxygen (3–5% oxygen) or atmospheric oxygen (21% oxygen) conditions. Tissue slices were maintained submerged in medium (floating), on Millicell Cell Culture inserts (Merck Millipore, PTFE, pore size 0.4 μm), on Alvetex Strata membranes (Reinnervate, polystyrene, pore size 5 μm, http://reinnervate.com/science-technical-resources/application-notes/maintenance-intact-tissues-using-alvetex-strata/), on Alvetex Polaris membranes (Reinnervate, pore size 0.4 μm) or loaded onto titanium grids and rotated using a tissue slice incubation unit^29^,^30^ (Alabama Research & Development, http://www.alspi.com/incubation.htm). An overview of cultivation conditions is shown in Table 1. Half of the medium was replenished after 24 hours. Slices were harvested at various time points and either snap-frozen for RNA isolation or fixed in 10% neutral buffered formalin for immunohistochemistry (IHC). Fixed slices were embedded in paraffin either in vertical or horizontal orientation as indicated in Figs 1 and 5.

### TMA preparation from thin tissue slices

Tissue slices were collected as FFPE blocks from all tumour samples and archived centrally at The Institute for Molecular Medicine Finland (FIMM) as tissue microarrays (TMA). The TMA archive is used for comparison of protein expression profiles across the various PREDECT consortium platforms by centralised IHC. TMAs were constructed by punching two 0.6 mm cores per sample from 30 vertically embedded tissue slices using a semi-automatic punching device (MiniCore). After arraying, we sectioned the TMA in 3.5 μm sections on Superfrost objective glasses (Kindler O Gmbh) using Microm 355S microtome (Thermo Scientific). Sections were dried and stored at +4°C. TMA sections were scanned at 0.22 micron resolution using Pannoramic P250 Flash whole slide scanner (3DHistech) equipped with 20× objective.

### Immunohistochemical staining

For examination of histopathology, paraffin sections (3–4 μm) were stained with hematoxylin and eosin. IHC staining was carried out by standard protocols. Briefly, sections were de-waxed in xylene and rehydrated in graded ethanol, heat mediated antigen retrieval of tissue sections was carried out before being allowed to cool. Specific antigen retrieval details for each tissue type are contained in Table 2. Endogenous peroxidases were blocked using 0.9–3% hydrogen peroxide for 10 minutes (Fisher), and non-specific antibody binding blocked by incubation with serum free blocking solution (Dako) or 10% normal serum block for 30 minutes. Tissue sections were then incubated with primary antibodies, before being probed with secondary antibodies (Table 2). Antibodies were visualised using 3,3′-diaminobenzidine (DAB) chromogen and counterstained with Meyer´s Hematoxylin (Dako) for 2 minutes. Sections were then dehydrated through graded alcohols, cleared in xylene and mounted.

### Quantitation of IHC

IHC scoring was carried out manually or using computer supported IHC quantification. Computer aided quantification was performed in whole tissue sections using the image analysis software Tissue Studio from Definiens or Aperio image analysis software (Aperio ePathology Solutions, Leica Biosystems). These programs allow quantification of the number of positively stained cells and the level of relative staining intensity in user defined regions of interest (ROI). Within these ROIs, algorithms were used that detect nuclei, membranous and cytoplasmic staining. Images were manually checked to ensure exclusion of mistakenly identified objects. Mean percentages of positively stained tumour cells from computer aided and manual IHC quantification were calculated across tumour types from at least three independent experiments. Statistical analysis was carried out using the student’s t test in GraphPad Prism (GraphPad Software). Spatial analysis of IHC images was carried out using bespoke in-house image processing algorithm for spatial feature detection in Matlab 13b (MathWorks). Slice images were compartmentalised arbitrarily into 50 layers, with each layer having an average thickness of 7.1 μm ± a standard deviation of 1.1 μm. ER H score was generated from ER staining quantified by image analysis software; it allows generation of a single figure that incorporates both the frequency of positively stained cells and the intensity of the staining. H scores were generated using the following formula: (1*per cent positive cells with intensity score 1) + (2*percent positive cells with intensity score 2) + (3*per cent positive cells with intensity score 3).

### Quantitation of necrosis in GEMM slices

Necrotic regions in *in vivo* tumours, 0 h, and cultivated slices were manually drawn after pathologist’s review. A graphics-editing program (Adobe Photoshop CS6, Adobe Systems) was used to draw the masks (i.e. ROI and tumour necrosis) on top of a downscaled (0.88 μm/pixel) versions of the original samples. During the process of drawing the masks, the full resolution whole-slide images were available in parallel to better discriminate the tissue morphology. The final tumour viability in the selected ROI was defined as a ratio of viable tumour tissue area in respect to the whole ROI area. The Matlab scientific computing environment was used to calculate the tumour viability measurements by importing the manually drawn image masks.

### Total RNA extraction from tumour tissues

Total RNA was extracted from snap-frozen tissue slices. Tissue lysates were prepared using lysing matrix D with the FastPrep sample preparation system (MP Biomedicals). Subsequently, RNA extraction was carried out using the RNeasy Mini kit (Qiagen) and on-column DNase (Qiagen) digestion was performed to eliminate genomic DNA contamination.

### cDNA synthesis and TaqMan-based qPCR

cDNA was generated using MMLV reverse transcriptase (Promega). Expression analysis was performed on the BioMark HD System (Fluidigm) according to manufacturer’s instructions. TaqMan assays were purchased from Applied Biosystems (Supplementary Table 1).

### Gene expression analysis

Gene expression qPCR data was normalised to a housekeeping gene and converted to log2 values. Differentially expressed genes were found using *limma* package in R statistics environment^31^. Tumour number was included in the linear model to take heterogeneity between tumours into account. Genes with FDR corrected p-value less than 0.05 and log-fold change of at least one were considered as significant.

Euclidean distance (square root of the sum of square differences) was used to measure the difference of the slice models from the *in vivo* situation. Before calculating Euclidean distances and doing Principal Component Analysis (PCA)^32^, the function *removeBatchEffect* from *limma* R package was used to remove the effect of the tumour number. PCA was done using *prcomp* function in base R. Genes containing any missing values were removed before calculating principal components. First two components (having largest and second largest variance, respectively) were shown on the scatterplot.

## Additional Information

**How to cite this article**: Davies, E. J. *et al*. Capturing complex tumour biology *in vitro*: histological and molecular characterisation of precision cut slices. *Sci. Rep*. **5**, 17187; doi: 10.1038/srep17187 (2015).

## Supplementary Material

Supplementary Information

## Figures and Tables

**Table 1:** Summary of tested cultivation conditions and tumour models used.

Support System	Oxygen Level	Models Tested
Floating/Unsupported	Low	CDX: MCF-7, NCI-H1437PDX: lung cancer LXFA 1647 (Oncotest), prostate cancer PC295GEMM: *Kras;Lkb1*
Floating/Unsupported	atmospheric	
Millicell filter support, 0.4 μm pore, non adherent (Millipore, #PICM 0RG 50/#PICM01250)	low	
Millicell filter support, 0.4 μm pore, non-adherent (Millipore, #PICM 0RG 50/#PICM01250)	atmospheric	
Strata filter support, 5 μm pore, adherent (Reinnervate)	atmospheric	CDX: MCF-7
Polaris filter support, 0.5 μm pore, adherent (Reinnervate)	atmospheric	
Tissue slice incubation unit, titanium grids (Alabama Research and Development)	atmospheric	CDX: MCF-7PDX: prostate cancer PC295GEMM: *Kras;Lkb1*
Floating/Unsupported	atmospheric	Primary patient: ER^+^ breast tumours
Millicell filter support, 0.4 μm pore, non-adherent (Millipore, #PICM 0RG 50/#PICM01250)	atmospheric	Primary patient: ER^+^ breast tumours and NSCLC tumours

**Table 2:** Tissue specific conditions for immunohistochemical staining (RT - room temperature).

Tissue Type	Primary Antibody	Retrieval	Secondary Antibody
MCF-7 CDX	Ki67 (MIB-1 clone, #M7240, Dako) 1:100, 60 mins, RT	pH6 antigen retrieval solution (Dako), 110 ^o^C at pressure for 2 mins	Envision+ anti-mouse secondary (Dako), 30 mins, RT
	Cleaved CK18 (M30 Cytodeath, #12140322001, Roche) 1:200, 60 mins, RT	pH6 antigen retrieval solution (Dako), 110 ^o^C at pressure for 5 mins	Envision+ anti-mouse secondary (Dako), 30 mins, RT
	ERα (ID5 clone, #M704701, Dako) 1:200, 60 mins, RT	pH6 antigen retrieval solution (Dako), 110 ^o^C at pressure for 2 mins	Biotinylated anti-mouse secondary (Dako) 30 mins, RT ABC amplification step (Vector Labs) 30 mins, RT
	HIF1α (#610959, BD Biosciences) 1:50, 120 mins, RT	pH8 EDTA solution, 110 ^o^C at pressure for 2 mins	Envision+ anti-mouse secondary (Dako), 30 mins, RT
	E-cadherin (24E10 clone, #3195, Cell Signaling Technology) 1:100, 60 mins, RT	pH6 antigen retrieval solution (Dako), 110 ^o^C at pressure for 2 mins	Biotinylated anti-rabbit secondary (Dako) 30 mins, RT ABC amplification step (Vector Labs) 30 mins, RT
	phospho-γH2AX (#2577, Cell Signaling Technology)	pH9 retrieval solution (Dako), 110 ^o^C at pressure for 2 mins	Envision+ anti-rabbit secondary (Dako), 30 mins, RT
	F4/80 (Serotec, #MCA497G) 1:200, 60 mins, RT	pH6 antigen retrieval solution (Dako), 110 ^o^C at pressure for 2 mins	Biotinylated anti-rat secondary (Dako) 30 mins, RT ABC amplification step (Vector Labs) 30 mins, RT
LungPDX and CDX	Ki67 (MIB-1 clone, #M7240, Dako) 1:75, 30 mins, RT	pH6 antigen retrieval solution (Dako), steam heat for 30 mins	pH6 antigen retrieval solution (Dako), steam heat for 30 mins
	Cleaved Caspase 3 (#9661, Cell Signaling) 1:75, 30 mins, RT	pH6 antigen retrieval solution (Dako), steam heat for 30 mins	Envision+ anti-mouse secondary (Dako), 30 mins, RT
Prostate PDX	Ki67 (MIB-1 clone, #M7240 Dako) 1:100, over night, 4°C	10 mM Na-citrate, pH6, at 99 ^o^C for 20	DAKO EnVision Detection Kit, Peroxidase/DAB, Rabbit/Mouse; 30 mins. RT, DAB 2 × 5 mins
	AR (#sp107, Cell Marque) 1:200, over night, 4°C	10 mM Na-citrate, pH6, at 99 ^o^C for 20	DAKO EnVision Detection Kit, Peroxidase/DAB, Rabbit/Mouse; 30 mins. RT, DAB 2 × 5 mins
