# Supplementary material for: Capturing complex tumour biology in vitro: histological and molecular characterisation of precision cut slices
Source: Sci Rep. 2015 Dec 9;5:17187. doi: 10.1038/srep17187 (PMC4673528; doi:10.1038/srep17187)
Supplement: Supplementary Information [file srep17187-s1.pdf]

Capturing complex tumour biology *in vitro*: histological and molecular characterisation of precision cut slices

Authors:

The PREDECT Consortium - Tissue Slice Platform*

*Emma J. Davies[1¤], Meng Dong[2¤], Matthias Gutekunst[2¤], Katja Närhi[3¤], Hanneke J. A. A. van Zoggel[4¤], Sami Blom[3], Taija M. af Hällström[3], Ashwini Nagaraj[3], Tauno Metsalu[5], Eva Oswald[6], Sigrun Erkens-Schulze[4], Juan A. Delgado San Martin[1], Riku Turkki[3], Stephen R. Wedge[1#], Julia Schueler[6#], Wytske van Weerden[4#], Emmy W Verschuren[3#†], Simon T. Barry[1#], Heiko van der Kuip[2#] and John A. Hickman[7†],

[†] Consortium Coordinators

[¤]Contributed equally to this work

[#]Senior Team Members

*www.predect.eu

[1]Oncology iMed, Bioscience, AstraZeneca, Alderley Park, Macclesfield, SK10 4TG, United Kingdom; [2]Institute for Molecular Medicine Finland (FIMM); [3]Dr Margarete Fischer-Bosch Institute of Clinical Pharmacology and University of Tuebingen, Auerbachstrasse 112, 70376 Stuttgart, Germany; University of Helsinki; Helsinki, Finland [4]Department of Urology, Erasmus MC Rotterdam, PO Box 2040, 3000 CA Rotterdam, The Netherlands; [5] Institute of Computer Science, University of Tartu, J. Liivi 2, 50409, Tartu, Estonia, [6] Oncotest GmbH, Am Flughafen 12-14, 79108 Freiburg, Germany; [7]Institut de Recherches Servier c/o 126 boulevard Pereire, 75017 Paris, France.

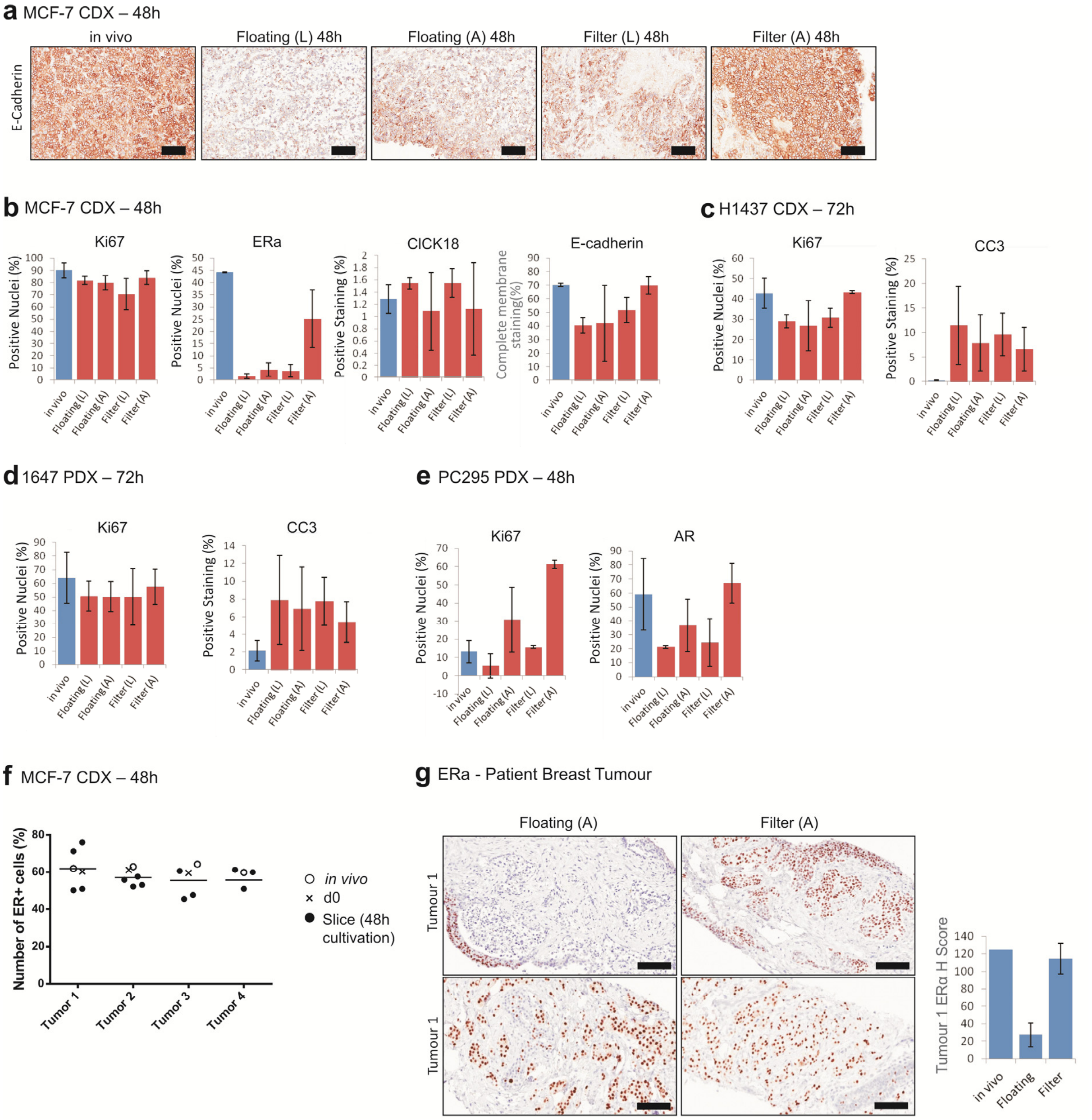

Supplementary Figure 1: **Changes in biomarkers according to culture condition.** (a) E-cadherin stained MCF-7 tumour tissue slice sections: retention of complete membranous staining is only observed in tumour slices cultivated on filter supports in atmospheric oxygen. Scale bars represent 100µm. (b-e) Histograms of the quantification of immunohistochemical staining for each of the biomarkers used to generate the heatmaps for each tumour type in Figure 2. Values reflect the means from three independent experiments, error bars represent standard deviations. (f) The total percentage of ER positive cells was quantified in the *in vivo* tumour, the day 0 (d0) samples and in slices cultivated for 48h. Levels of ER positive cells were similar to that found *in vivo.* (g) Slices from two patient breast tumours were cultivated with and without a filter support. Cultivation without a filter support caused a significant loss of ERα positive cells in slices from tumour 1 but had no effect on ERα positive cells in slices from tumour 2. Scale bars represent 100µm.

a

oxygen level		A	L	A	L		A	L	A	L
filter-support	d0	+	+	-	-	d0	+	+	-	-
breast MCF-7 CDX	0	6	4	nd	nd	0	22	16	nd	nd
		upregulated					downregulated			

b MCF-7 CDX

oxygen level	A	L	A	L	A	L	A	L
filter-support	+	+	-	-	+	+	-	-
apoptosis	1	0	nd	nd	4	1	nd	nd
cell cycle	0	2	nd	nd	10	6	nd	nd
DDR	1	0	nd	nd	5	4	nd	nd
necrosis/autophagy	0	0	nd	nd	0	0	nd	nd
NFkB	0	0	nd	nd	1	2	nd	nd
p38/JNK	0	0	nd	nd	2	1	nd	nd
ROS	2	0	nd	nd	0	1	nd	nd
UPR/ER stress	2	2	nd	nd	0	1	nd	nd

c MCF-7 CDX – Filter, Low Oxygen

Horizontal section Vertical section

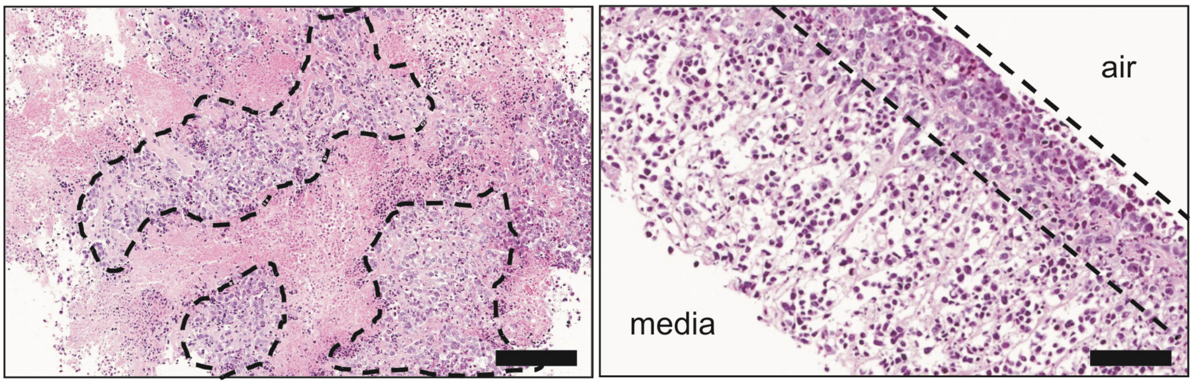

Supplementary Figure 2: **Stress biomarker changes in MCF-7 tumour slices.** (a) Total numbers of differentially expressed stress transcripts in MCF-7 tumour slices cultivated on filters in atmospheric and low oxygen conditions. Insufficient RNA could be extracted from MCF-7 tumour slices cultivated under floating conditions. (b) Table of the numbers of differentially expressed genes (DEGs) and their associated functions in MCF-7 tumour slices. Results are from three independent experiments. (c) H&E stained sections of MCF-7 tumour slices cultivated on filters under conditions of low oxygen; dashed line indicates areas of viable cells. Scale bars represent 100µm.

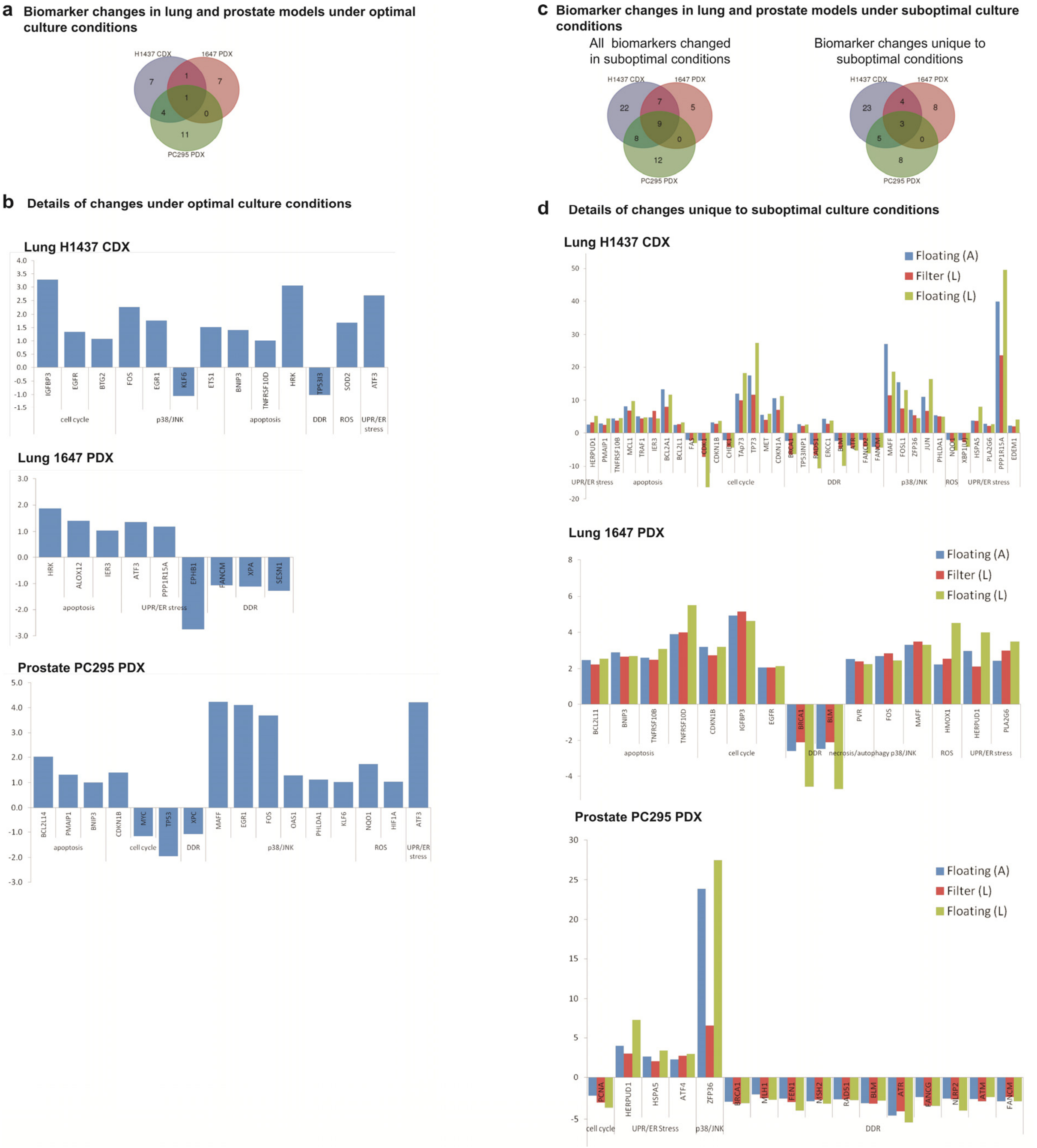

Supplementary Figure 3: **Stress biomarker changes for each model cultivated under either optimal or suboptimal conditions.** The fold-changes in transcriptional biomarkers observed in slices from MCF-7, PC295, H1437 and LXFA 1647 xenografts after cultivation. Red indicates significantly up-regulated genes and green indicates down-regulated genes (p<0.05). (a) Venn diagram showing the stress gene transcripts deregulated in three xenograft models. (b) Details of the individual changes shown in the Venn diagram. (c) Venn diagrams of all biomarker changes observed under suboptimal conditions (floating and low oxygen conditions) and those unique to suboptimal conditions of slice culture. (d) Details of the individual changes in slices from three xenograft models unique to suboptimal conditions. Results are from three independent experiments.

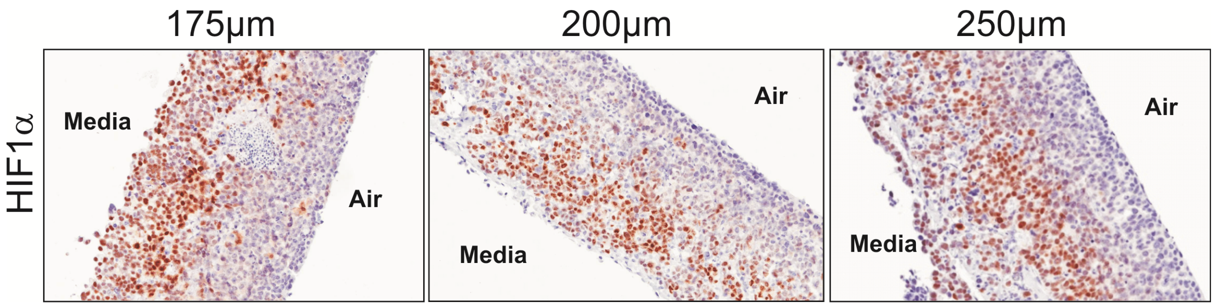

Supplementary Figure 4: **HIF1α expression in slices of varying thicknesses.** Generation of a gradient of HIF1α expression after 24h in slices of MCF-7 CDX cut at 175 to 250µm and incubated under optimal conditions (filter support, incubation in air, on Millicell Cell Culture inserts (see Methods).

Supplementary Table 1: **A list of the transcripts analysed and their functional classification.**

Gene names	Assay (Applied)	Functional Gene Grouping	Upstream pathway/transcrition factor	Refs #
**ABCB1**	**Hs00184500_m1**	**Drug transporter**	p53/NFBkB	[1]
**ACTB**	**Hs03023943_g1**	**housekeeping gene**		
**AIFM2**	**Hs01097299_m1**	**apoptosis**	p53	[2]
**ALOX12**	**Hs00911144_m1**	**apoptosis**	NFkB	[3]
**APAF1**	**Hs00559440_m1**	**apoptosis**	p53	[4]
**ATF2**	**Hs01099617_m1**	**UPR/ER stress**	target of stress regulated miR-451	[5]
**ATF3**	**Hs00231069_m1**	**UPR/ER stress**	p53/p38	[6]
**ATF4**	**Hs00909569_g1**	**UPR/ER Stress**	UPR target gene	[7]
**ATF6**	**Hs00232586_m1**	**UPR/ER Stress**	UPR transcrition factor	[8]
**ATG12**	**Hs01047860_g1**	**necrosis/autophagy**	taget of ATF4	[9]
**ATG5**	**Hs01555465_m1**	**necrosis/autophagy**	induced by DNA damage	[10]
**ATG7**	**Hs00197348_m1**	**necrosis/autophagy**	target of miR17	[11]
**ATM**	**Hs00175892_m1**	**DNA damage and repair**	BRCA1/E2F1/CtIP	[12]
**ATR**	**Hs00992132_m1**	**DNA damage and repair**	target of stress regulated miR-185	[13]
**BAK1**	**Hs00832876_g1**	**apoptosis**	p53	[14]
**BCL2**	**Hs01048932_g1**	**apoptosis**	NFkB/p53	[15, 16]
**BCL2A1**	**Hs03405589_m1**	**apoptosis**	p53/NFkB	[17, 18]
**BCL2L1**	**Hs01067345_g1**	**apoptosis**	NFkB	[19]
**BCL2L11**	**Hs01083836_m1**	**apoptosis**	NFkB	[20]
**BCL2L13**	**Hs00209787_m1**	**apoptosis**	prosurvival signals and oncogenes	
**BCL2L14**	**Hs00373302_m1**	**apoptosis**	p53	[21]
**BECN1**	**Hs01011594_g1**	**necrosis/autophagy**	NFkB	[22]
**BID**	**Hs00609632_m1**	**apoptosis**	p53	[23]
**BIK**	**Hs00154189_m1**	**apoptosis**	DNA damage/cytokines	[24]
**BIRC2**	**Hs01112284_m1**	**apoptosis**	NFkB	[25]
**BLM**	**Hs01119886_g1**	**DNA damage and repair**	prosurvival signals and oncogenes	[26]
**BNIP3**	**Hs00969289_m1**	**apoptosis**	NFkB	[27]
**BOK**	**Hs00261296_m1**	**apoptosis**	p53	[28]
**BRCA1**	**Hs01556185_m1**	**DNA damage and repair**	regulated by specific factors binding single-stranded DNA	[29]
**BTG2**	**Hs00198887_m1**	**proliferation/cell cycle regulation**	p53	[17]

**CCNA2**	**Hs00153138_m1**	**proliferation/cell cycle regulation**	p53	[30]
**CCNB1**	**Hs01030097_m1**	**proliferation/cell cycle regulation**	p53	[31]
**CCNG1**	**Hs00171112_m1**	**proliferation/cell cycle regulation**	p53	[32]
**CCNK**	**Hs01083210_m1**	**proliferation/cell cycle regulation**	p53	[33]
**CDC25C**	**Hs00156411_m1**	**proliferation/cell cycle regulation**	p53	[34]
**CDK1**	**Hs00364293_m1**	**proliferation/cell cycle regulation**	p53	[35]
**CDKN1A**	**Hs99999142_m1**	**proliferation/cell cycle regulation**	p53/NFkB	[17,36]
**CDKN1B**	**Hs00153277_m1**	**proliferation/cell cycle regulation**	prosurvival signals and oncogenes	[37]
**CFLAR**	**Hs01116281_m1**	**apoptosis**	NFkB	[38]
**CHEK1**	**Hs00967510_g1**	**proliferation/cell cycle regulation**	NF-Y-p53	[39]
**DDB2**	**Hs03044953_m1**	**DNA damage and repair**	p53/BRCA1/AP1	[17]
**DNAJC3**	**Hs00534489_m1**	**UPR/ER Stress**	UPR target gene	[40]
**EDEM1**	**Hs00976004_m1**	**UPR/ER Stress**	UPR target gene	[40]
**EDEM2**	**Hs01076556_m1**	**UPR/ER Stress**	UPR target gene	[41]
**EGFR**	**Hs01076078_m1**	**proliferation/cell cycle regulation**	p53/NFBkB	[42,16]
**EGR1**	**Hs00152928_m1**	**p38/JNK**	NFkB/p38	[43,44]
**EPHB1**	**Hs01057855_m1**	**UPR/ER Stress**	UPR target gene	
**ERCC1**	**Hs01012156_m1**	**DNA damage and repair**	AP-1/MZF1	[45]
**ETS1**	**Hs00901423_m1**	**p38/JNK**	p38	[46]
**FANCA**	**Hs01116661_m1**	**DNA damage and repair**	target of miR-503	[47]
**FANCD2**	**Hs00395700_m1**	**DNA damage and repair**	transcriptionally regulated under stress (hypoxia)	[48]
**FANCG**	**Hs00184947_m1**	**DNA damage and repair**	target of stress responsive miR23a	[49]
**FANCM**	**Hs00913609_m1**	**DNA damage and repair**	HOXC9	[13]
**FAS**	**Hs00531110_m1**	**apoptosis**	p53/NFBkB	[17,50]
**FASLG**	**Hs00181226_g1**	**apoptosis**	p53/NFBkB	[51]
**FEN1**	**Hs01099393_g1**	**DNA damage and repair**	p53/AP1	[52]
**FOS**	**Hs01119266_g1**	**p38/JNK**	p53/p38	[44]
**FOSL1**	**Hs04187685_m1**	**p38/JNK**	p38	[44]
**FTH1**	**Hs01000476_g1**	**ROS**	NFkB	[53]

**GRB2**	**Hs00257910_s1**	**necrosis/autophagy**		
**GSTP1**	**Hs00168310_m1**	**ROS**	NFkB	[54]
**HERPUD1**	**Hs01124265_m1**	**UPR/ER Stress**	UPR target gene	[40]
**HGF**	**Hs00900067_m1**	**proliferation/cell cycle regulation**	p53/NFBkB	[17,55]
**HIF1A**	**Hs00936368_m1**	**ROS**	NFkB/transcriptionally regulated under hypoxia	[56,57]
**HMOX1**	**Hs01110250_m1**	**ROS**	NFkB	[58]
**HRK**	**Hs01388767_g1**	**apoptosis**	prosurvival signals and oncogenes	[59]
**HSP90AA1**	**Hs00743767_sH**	**UPR/ER Stress**	NFkB	[60]
**HSPA5**	**Hs00946084_g1**	**UPR/ER Stress**	UPR target gene	[40]
**IER3**	**Hs04187506_g1**	**apoptosis**	p53/NFBkB	[16,61]
**IGFBP3**	**Hs00426287_m1**	**proliferation/cell cycle regulation**	p53	[16]
**JUN**	**Hs00277190_s1**	**p38/JNK**	p38	[44]
**JUND**	**Hs02330233_u1**	**p38/JNK**	p38	[62]
**KLF6**	**Hs00296661_s1**	**p38/JNK**	p38	[44]
**MAFF**	**Hs00202412_m1**	**p38/JNK**	p38	[44]
**MAPK8**	**Hs01548508_m1**	**p38/JNK**	KLF4/FOXM1	[63]
**MCL1**	**Hs00172036_m1**	**apoptosis**	prosurvival signals and oncogenes	[64]
**MDM2**	**Hs00234753_m1**	**proliferation/cell cycle regulation**	p53	[17]
**MDM4**	**Hs00159092_m1**	**proliferation/cell cycle regulation**	p53	[17]
**MET**	**Hs01565584_m1**	**proliferation/cell cycle regulation**	p53	[16]
**MLH1**	**Hs00179866_m1**	**DNA damage and repair**	p53/AP1	[17]
**MSH2**	**Hs00954125_m1**	**DNA damage and repair**	p53/AP1	[16]
**MYC**	**Hs00905030_m1**	**proliferation/cell cycle regulation**	p53/NFBkB/p38	[17,65,44]
**NLRP2**	**Hs01546932_m1**	**DNA damage and repair**	NFkB	[66]
**NOS2A**	**Hs01075523_m1**	**apoptosis**	NFkB	[67]
**NQO1**	**Hs00168547_m1**	**ROS**	NFkB	[68]
**OAS1**	**Hs00242943_m1**	**p38/JNK**	p38	[44]
**P2RY13**	**Hs01090437_g1**	**UPR/ER Stress**	UPR target gene	
**PARP1**	**Hs00911377_g1**	**necrosis/autophagy**		
**PCNA**	**Hs00696862_m1**	**proliferation/cell cycle regulation**	p53/AP1	[17]

**PGK1**	**Hs00943178_g1**	**housekeeping gene**		
**PHLDA1**	**Hs00378285_g1**	**p38/JNK**	p38	44
**PIDD**	**Hs01076855_g1**	**apoptosis**	p53	69
**PLA2G6**	**Hs00895666_m1**	**UPR/ER Stress**	UPR target gene	70
**PMAIP1**	**Hs00560402_m1**	**apoptosis**	p53	17
**POL2RB**	**Hs00265358_m1**	**housekeeping gene**		
**PPP1R15A**	**Hs00921550_g1**	**UPR/ER Stress**	UPR target gene	71
**PRDX1**	**Hs03044568_g1**	**ROS**	NRF-2 upon oxidative stress	72
**PVR**	**Hs00197846_m1**	**necrosis/autophagy**	NRF-1 upon oxidative stress	73
**RAD51**	**Hs00947964_m1**	**DNA damage and repair**	transcriptionally regulated under stress (hypoxia)	48
**RB1**	**Hs01078075_m1**	**proliferation/cell cycle regulation**	p53	16
**RIPK1**	**Hs00169407_m1**	**necrosis/autophagy**		
**RPA1**	**Hs01556877_m1**	**proliferation/cell cycle regulation**		
**RPLP0**	**Hs02992885_s1**	**housekeeping gene**		
**RRM2B**	**Hs00968427_m1**	**DNA damage and repair**	p53	74
**SENP2**	**Hs00989703_m1**	**apoptosis**	NFkB	75
**SESN1**	**Hs00902782_m1**	**DNA damage and repair**	p53	17
**SFN**	**Hs00968567_s1**	**proliferation/cell cycle regulation**	p53	76
**SHISA5**	**Hs00429977_m1**	**apoptosis**	p53	77
**SIRT1**	**Hs01009000_m1**	**DNA damage and repair**	target of stress inducible miR34a and HuR	78
**SOD1**	**Hs00916176_m1**	**ROS**	NFkB	79
**SOD2**	**Hs04260076_g1**	**ROS**	NFkB	80
**SQSTM1**	**Hs01061917_g1**	**ROS**	NRF-2 upon oxidative stress	81
**SUMO1**	**Hs00830844_g1**	**DNA damage and repair**	target of stress inducible miR150	
**TAP73**	**Hs01056228_m1**	**proliferation/cell cycle regulation**	p53	82
**TBP**	**Hs00427621_m1**	**housekeeping gene**		
**TERT**	**Hs00972656_m1**	**proliferation/cell cycle regulation**	p53/NFBkB	83
**TNFRSF10A**	**Hs00269492_m1**	**apoptosis**	p53/NFBkB	84
**TNFRSF10B**	**Hs00366278_m1**	**apoptosis**	p53/NFBkB	17
**TNFRSF10C**	**Hs00182570_m1**	**apoptosis**	p53/NFBkB	85

**TNFRSF10D**	**Hs04187502_m1**	**apoptosis**	p53/NFBkB	[17]
**TP53**	**Hs99999147_m1**	**proliferation/cell cycle regulation**	p53/NFBkB	[86, 16]
**TP53AIP1**	**Hs00986095_m1**	**apoptosis**	p53/NFBkB	[87]
**TP53I3**	**Hs00936520_m1**	**DNA damage and repair**	p53/NFBkB	[17]
**TP53INP1**	**Hs00264502_m1**	**DNA damage and repair**	p53/NFBkB	[17]
**TP63**	**Hs00978342_m1**	**proliferation/cell cycle regulation**	p53/NFBkB	[16]
**TP73**	**Hs01056230_m1**	**proliferation/cell cycle regulation**	p53/NFBkB	[17]
**TRAF1**	**Hs01090167_g1**	**apoptosis**	p53/NFBkB	[88]
**TRAF2**	**Hs00184192_m1**	**apoptosis**	p53/NFBkB	[89]
**TRPM3**	**Hs01558432_m1**	**p38/JNK**		
**TXN**	**Hs00828652_m1**	**ROS**	NRF-2 upon oxidative stress	[90]
**XBP1**	**Hs00231936_m1**	**UPR/ER Stress**	UPR target gene	[91]
**XBP1(U)**	**Hs02856596_m1**	**UPR/ER Stress**	UPR target gene	[91]
**XIAP**	**Hs00745222_s1**	**apoptosis**	NFkB	[92]
**XPA**	**Hs00166045_m1**	**DNA damage and repair**	HIF1a	[57]
**XPC**	**Hs01104213_m1**	**DNA damage and repair**	p53/BRCA1/AP1	[17]
**XRCC1**	**Hs00959834_m1**	**DNA damage and repair**	E2F1/AP1	[93]
**XRCC3**	**Hs00193725_m1**	**DNA damage and repair**	transcr inducable by irradiation	[94]
**ZFP36**	**Hs00185658_m1**	**p38/JNK**	p38	[44]
**ZMAT3**	**Hs00536976_m1**	**apoptosis**	p53	[95]

Supplementary Table 2: **Part 1: The fold-changes in transcriptional biomarkers observed in slices from H1437 after cultivation.**

H1437 lung CDX

filter, atmospheric oxygen vs in vivo

process	ID	logFC	P.Value	adj.P.Val
apoptosis	HRK	3.06	2.21E-03	1.91E-02
apoptosis	BNIP3	1.41	5.53E-04	6.09E-03
apoptosis	TNFRSF10D	1.00	1.83E-04	3.16E-03
cell cycle	IGFBP3	3.29	1.08E-03	1.09E-02
cell cycle	EGFR	1.35	4.91E-03	3.64E-02
cell cycle	BTG2	1.09	7.47E-03	4.76E-02
DDR	TP53I3	-1.02	5.11E-03	3.64E-02
p38/JNK	FOS	2.26	2.38E-06	1.44E-04
p38/JNK	EGR1	1.76	3.43E-05	1.06E-03
p38/JNK	ETS1	1.52	6.18E-05	1.25E-03
p38/JNK	KLF6	-1.06	3.79E-05	1.06E-03
ROS	SOD2	1.69	4.37E-05	1.06E-03
UPR/ER str	ATF3	2.70	2.36E-07	2.85E-05

filter, low oxygen vs in vivo

process	ID	logFC	P.Value	adj.P.Val
apoptosis	BCL2A1	3.01	1.16E-03	2.86E-03
apoptosis	MCL1	2.76	1.12E-07	1.23E-06
apoptosis	IER3	2.72	7.86E-04	2.21E-03
apoptosis	TNFRSF10D	2.51	1.01E-08	2.05E-07
apoptosis	BNIP3	2.23	7.72E-06	4.45E-05
apoptosis	TRAF1	2.12	8.58E-05	3.35E-04
apoptosis	TNFRSF10B	1.88	6.87E-08	8.35E-07
apoptosis	BCL2L1	1.39	1.39E-03	3.16E-03
apoptosis	PMAIP1	1.29	3.59E-06	2.29E-05
apoptosis	XIAP	1.28	8.55E-06	4.50E-05
apoptosis	TNFRSF10C	1.04	4.07E-03	8.48E-03
apoptosis	FAS	-1.11	7.57E-03	1.40E-02
apoptosis	ZMAT3	-1.14	1.06E-03	2.71E-03
cell cycle	IGFBP3	4.36	9.71E-05	3.56E-04
cell cycle	TP73	3.55	2.69E-04	8.33E-04
cell cycle	TAp73	3.32	1.19E-03	2.88E-03
cell cycle	BTG2	3.27	3.68E-07	3.18E-06
cell cycle	EGFR	2.84	8.32E-06	4.50E-05
cell cycle	CDKN1A	2.81	1.17E-06	8.31E-06
cell cycle	MET	1.96	1.96E-07	1.82E-06
cell cycle	CDKN1B	1.49	1.04E-06	7.88E-06
cell cycle	TP63	1.25	1.14E-02	1.99E-02
cell cycle	PCNA	-1.56	2.09E-04	6.82E-04
cell cycle	CDC25C	-1.71	6.22E-04	1.84E-03
cell cycle	CCNA2	-1.84	1.30E-03	3.03E-03
cell cycle	CCNB1	-1.89	1.93E-04	6.65E-04
cell cycle	CHEK1	-2.06	1.12E-04	3.97E-04
cell cycle	HGF	-2.21	2.55E-02	4.17E-02
cell cycle	CDK1	-2.84	4.23E-06	2.56E-05
DDR	ERCC1	1.44	8.76E-04	2.36E-03
DDR	TP53INP1	1.07	3.18E-03	6.75E-03
DDR	MSH2	-1.08	1.98E-04	6.65E-04
DDR	SESN1	-1.09	1.21E-05	5.84E-05
DDR	XRCC3	-1.93	2.22E-04	7.06E-04
DDR	FANCD2	-2.01	1.20E-05	5.84E-05
DDR	ATR	-2.15	6.90E-08	8.35E-07
DDR	BLM	-2.24	8.92E-05	3.37E-04
DDR	FANCM	-2.26	1.87E-07	1.82E-06
DDR	BRCA1	-2.68	5.57E-07	4.50E-06
DDR	RAD51	-2.73	6.31E-05	2.63E-04
necrosis/au	PARP1	-1.00	5.35E-03	1.08E-02
p38/JNK	ETS1	4.72	1.72E-10	1.04E-08
p38/JNK	EGR1	4.02	3.53E-09	1.43E-07
p38/JNK	FOS	3.84	5.40E-09	1.63E-07
p38/JNK	MAFF	3.52	2.69E-05	1.21E-04
p38/JNK	FOSL1	2.89	1.07E-03	2.71E-03
p38/JNK	JUN	2.73	6.93E-04	2.00E-03
p38/JNK	ZFP36	2.42	4.61E-03	9.45E-03
p38/JNK	PHLDA1	2.34	2.17E-06	1.46E-05
p38/JNK	KLF6	1.93	5.53E-08	8.35E-07
ROS	SOD2	3.67	8.25E-09	2.00E-07
ROS	NQO1	-1.41	7.61E-03	1.40E-02
UPR/ER str	ATF3	5.94	1.76E-11	2.13E-09
UPR/ER Str	PPP1R15A	4.56	4.71E-05	2.04E-04
UPR/ER Str	HSPA5	1.87	1.41E-08	2.44E-07
UPR/ER Str	HERPUD1	1.65	2.44E-05	1.13E-04
UPR/ER Str	PLA2G6	1.04	1.77E-02	3.01E-02
UPR/ER Str	EDEM1	1.02	8.56E-05	3.35E-04
UPR/ER Str	XBP1	-1.36	2.69E-03	5.80E-03
UPR/ER Str	XBP1(U)	-1.44	1.68E-03	3.76E-03

floating, atmospheric oxygen vs in vivo

process	ID	logFC	P.Value	adj.P.Val
apoptosis	BCL2A1	3.74	3.86E-04	1.20E-03
apoptosis	MCL1	3.03	3.78E-08	5.08E-07
apoptosis	TNFRSF10D	2.83	2.32E-09	9.36E-08
apoptosis	BNIP3	2.81	6.33E-07	4.50E-06
apoptosis	TRAF1	2.31	3.69E-05	1.59E-04
apoptosis	IER3	2.24	3.34E-03	7.93E-03
apoptosis	BOK	2.16	1.85E-02	3.20E-02
apoptosis	TNFRSF10B	2.11	1.79E-08	2.71E-07
apoptosis	PMAIP1	1.51	6.07E-07	4.50E-06
apoptosis	BCL2L1	1.30	2.30E-03	5.67E-03
apoptosis	BIK	1.06	2.44E-02	3.94E-02
apoptosis	BAK1	1.01	2.09E-02	3.46E-02
apoptosis	FAS	-1.07	9.06E-03	1.78E-02
cell cycle	IGFBP3	5.52	9.58E-06	5.52E-05
cell cycle	TP73	4.13	7.13E-05	2.70E-04
cell cycle	TAp73	3.59	6.48E-04	1.91E-03
cell cycle	CDKN1A	3.41	1.33E-07	1.46E-06
cell cycle	EGFR	2.86	7.62E-06	4.61E-05
cell cycle	MET	2.44	1.53E-08	2.71E-07
cell cycle	BTG2	2.19	2.60E-05	1.31E-04
cell cycle	CDKN1B	1.66	3.01E-07	2.92E-06
cell cycle	CHEK1	-1.15	9.25E-03	1.78E-02
cell cycle	CDK1	-1.23	5.92E-03	1.26E-02
cell cycle	HGF	-1.64	2.70E-02	4.18E-02
DDR	ERCC1	2.10	2.97E-05	1.38E-04
DDR	TP53INP1	1.40	4.14E-04	1.25E-03
DDR	SIRT1	1.22	1.09E-04	3.76E-04
DDR	FANCD2	-1.04	3.50E-03	8.14E-03
DDR	BRCA1	-1.27	8.15E-04	2.34E-03
DDR	BLM	-1.28	7.12E-03	1.46E-02
DDR	RAD51	-1.75	4.95E-03	1.11E-02
DDR	ATR	-1.88	3.14E-07	2.92E-06
DDR	FANCM	-2.06	5.37E-07	4.33E-06
necrosis/au	PVR	1.99	5.67E-03	1.23E-02
necrosis/au	ATG5	1.20	1.85E-05	9.71E-05
necrosis/au	ATG7	1.07	6.02E-05	2.43E-04
p38/JNK	MAFF	4.76	1.16E-06	7.78E-06
p38/JNK	ETS1	4.70	1.83E-10	1.11E-08
p38/JNK	FOSL1	3.96	7.11E-05	2.70E-04
p38/JNK	EGR1	3.65	1.11E-08	2.70E-07
p38/JNK	JUN	3.47	8.70E-05	3.09E-04
p38/JNK	FOS	3.18	5.09E-08	6.15E-07
p38/JNK	ZFP36	2.80	1.64E-03	4.22E-03
p38/JNK	PHLDA1	2.42	1.53E-06	9.75E-06
p38/JNK	KLF6	1.59	5.01E-07	4.33E-06
p38/JNK	MAPK8	1.06	1.37E-02	2.48E-02
ROS	SOD2	3.44	1.78E-08	2.71E-07
ROS	SQSTM1	1.47	1.09E-03	3.01E-03
ROS	HMOX1	1.29	2.00E-02	3.36E-02
ROS	NQO1	-1.08	3.15E-02	4.82E-02
ROS	HIF1A	-1.22	3.32E-05	1.49E-04
UPR/ER str	PPP1R15A	5.32	1.01E-05	5.58E-05
UPR/ER str	ATF3	5.22	8.82E-11	1.07E-08
UPR/ER str	HSPA5	1.92	1.06E-08	2.70E-07
UPR/ER str	PLA2G6	1.44	2.46E-03	5.96E-03
UPR/ER str	HERPUD1	1.36	1.55E-04	5.22E-04
UPR/ER str	EDEM1	1.13	2.95E-05	1.38E-04
UPR/ER str	XBP1(U)	-1.12	9.12E-03	1.78E-02

floating, low oxygen vs in vivo

process	ID	logFC	P.Value	adj.P.Val
apoptosis	BCL2A1	3.55	4.90E-04	9.72E-04
apoptosis	MCL1	3.29	1.42E-08	1.71E-07
apoptosis	TNFRSF10D	3.14	6.63E-10	2.01E-08
apoptosis	BNIP3	3.12	1.96E-07	1.25E-06
apoptosis	TRAF1	2.25	4.88E-05	1.34E-04
apoptosis	TNFRSF10B	2.16	1.35E-08	1.71E-07
apoptosis	IER3	2.14	4.48E-03	6.69E-03
apoptosis	PMAIP1	2.12	1.23E-08	1.71E-07
apoptosis	XIAP	1.74	2.95E-07	1.79E-06
apoptosis	BCL2L1	1.69	2.73E-04	5.60E-04
apoptosis	TNFRSF10C	1.11	2.50E-03	4.03E-03
apoptosis	BID	-1.36	4.44E-03	6.69E-03
apoptosis	ZMAT3	-1.50	1.05E-04	2.49E-04
apoptosis	FAS	-1.59	5.87E-04	1.11E-03
cell cycle	IGFBP3	5.94	4.41E-06	1.69E-05
cell cycle	TP73	4.78	1.85E-05	6.06E-05
cell cycle	TAp73	4.19	1.84E-04	3.90E-04
cell cycle	EGFR	3.58	6.83E-07	3.44E-06
cell cycle	CDKN1A	3.50	9.89E-08	7.04E-07
cell cycle	BTG2	2.91	1.38E-06	6.40E-06
cell cycle	MET	2.52	1.07E-08	1.71E-07
cell cycle	TP63	1.91	5.88E-04	1.11E-03
cell cycle	CDKN1B	1.87	7.50E-08	6.48E-07
cell cycle	RB1	1.21	4.42E-06	1.69E-05
cell cycle	MDM4	1.05	1.76E-04	3.80E-04
cell cycle	MYC	-1.53	2.56E-03	4.08E-03
cell cycle	PCNA	-1.65	1.30E-04	2.97E-04
cell cycle	CHEK1	-2.12	8.64E-05	2.13E-04
cell cycle	CDC25C	-2.41	2.80E-05	8.47E-05
cell cycle	CCNB1	-2.78	4.48E-06	1.69E-05
cell cycle	CCNA2	-2.93	2.08E-05	6.45E-05
cell cycle	CDK1	-4.04	8.22E-08	6.63E-07
DDR	ERCC1	1.92	7.25E-05	1.91E-04
DDR	TP53INP1	1.33	6.43E-04	1.20E-03
DDR	DDB2	1.01	6.90E-03	1.01E-02
DDR	MSH2	-1.33	2.90E-05	8.56E-05
DDR	XRCC3	-1.56	1.26E-03	2.21E-03
DDR	SESN1	-1.61	1.78E-07	1.20E-06
DDR	FANCA	-2.11	4.98E-04	9.73E-04
DDR	FANCM	-2.14	3.48E-07	2.00E-06
DDR	ATR	-2.38	2.04E-08	2.06E-07
DDR	FANCD2	-2.61	7.44E-07	3.60E-06
DDR	BRCA1	-2.65	6.42E-07	3.38E-06
DDR	BLM	-3.30	1.80E-06	8.05E-06
DDR	RAD51	-3.41	8.63E-06	2.90E-05
necrosis/au	PVR	1.85	8.79E-03	1.21E-02
necrosis/au	ATG5	1.37	4.68E-06	1.71E-05
necrosis/au	ATG12	1.16	8.88E-05	2.15E-04
necrosis/au	ATG7	1.14	3.49E-05	1.01E-04
necrosis/au	RIPK1	1.02	5.02E-05	1.35E-04
necrosis/au	PARP1	-1.82	4.34E-05	1.22E-04
p38/JNK	ETS1	5.53	2.43E-11	1.47E-09
p38/JNK	MAFF	4.22	4.18E-06	1.69E-05
p38/JNK	JUN	4.04	1.98E-05	6.31E-05
p38/JNK	EGR1	3.85	6.05E-09	1.22E-07
p38/JNK	FOSL1	3.71	1.28E-04	2.97E-04
p38/JNK	FOS	3.45	1.94E-08	2.06E-07
p38/JNK	KLF6	2.36	5.15E-09	1.22E-07
p38/JNK	PHLDA1	2.30	2.61E-06	1.13E-05
p38/JNK	ZFP36	2.18	8.70E-03	1.21E-02
p38/JNK	MAPK8	1.15	8.46E-03	1.20E-02
ROS	SOD2	2.98	9.67E-08	7.04E-07
ROS	SQSTM1	1.22	4.07E-03	6.23E-03
ROS	PRDX1	-1.06	1.83E-03	3.00E-03
ROS	SOD1	-1.06	1.67E-03	2.80E-03
ROS	HIF1A	-1.40	8.63E-06	2.90E-05
ROS	GSTP1	-1.63	1.57E-03	2.71E-03
ROS	NQO1	-2.38	1.44E-04	3.22E-04
UPR/ER str	ATF3	6.14	1.17E-11	1.41E-09
UPR/ER str	PPP1R15A	5.63	5.59E-06	1.99E-05
UPR/ER str	HSPA5	3.02	4.25E-11	1.71E-09
UPR/ER str	HERPUD1	2.37	5.20E-07	2.86E-06
UPR/ER str	EDEM1	2.00	6.19E-08	5.76E-07
UPR/ER str	PLA2G6	1.41	2.86E-03	4.49E-03
UPR/ER str	ATF4	1.30	8.48E-05	2.13E-04
UPR/ER str	XBP1	-1.77	3.57E-04	7.20E-04
UPR/ER str	XBP1(U)	-2.06	8.37E-05	2.13E-04

Red indicates significantly up-regulated genes, and green indicates down-regulated genes ($p < 0.05$).
The different stress pathways are indicated by different colors.

Supplementary Table 2: **Part 2: The fold-changes in transcriptional biomarkers observed in slices from 1647 lung PDX after cultivation.**

1647 lung PDX

filter, atmospheric oxygen vs in vivo

process	ID	logFC	P.Value	adj.P.Val
apoptosis	HRK	1.87	2.77E-05	4.12E-04
apoptosis	ALOX12	1.41	9.71E-06	1.93E-04
apoptosis	IER3	1.03	1.37E-04	1.36E-03
DDR	FANCM	-1.06	8.13E-06	1.93E-04
DDR	XPA	-1.10	6.15E-05	6.65E-04
DDR	SESN1	-1.26	1.66E-05	2.83E-04
UPR/ER stress	ATF3	1.37	1.44E-07	1.71E-05
UPR/ER Stress	PPP1R15A	1.19	5.41E-06	1.93E-04
UPR/ER Stress	EPHB1	-2.76	8.01E-06	1.93E-04

filter, low oxygen vs in vivo

process	ID	logFC	P.Value	adj.P.Val
apoptosis	HRK	2.13	6.05E-06	4.24E-05
apoptosis	TNFRSF10D	1.99	1.24E-09	3.68E-08
apoptosis	ALOX12	1.95	1.35E-07	2.30E-06
apoptosis	IER3	1.81	1.27E-07	2.30E-06
apoptosis	BNIP3	1.41	8.46E-07	8.39E-06
apoptosis	TNFRSF10B	1.32	6.19E-07	6.70E-06
apoptosis	BCL2L11	1.16	1.45E-05	7.91E-05
cell cycle	IGFBP3	2.36	8.48E-11	5.05E-09
cell cycle	CDKN1B	1.45	1.27E-06	1.16E-05
cell cycle	MET	1.37	5.09E-07	6.06E-06
cell cycle	CDKN1A	1.12	9.49E-06	5.94E-05
cell cycle	TP73	1.04	7.66E-05	2.94E-04
cell cycle	EGFR	1.04	1.32E-05	7.86E-05
DDR	BLM	-1.06	1.72E-05	8.51E-05
DDR	BRCA1	-1.07	2.76E-06	2.34E-05
DDR	SESN1	-1.26	1.61E-05	8.31E-05
DDR	FANCM	-1.45	1.22E-07	2.30E-06
necrosis/autoph.	PVR	1.27	7.49E-06	4.95E-05
p38/JNK	MAFF	1.80	4.55E-07	6.01E-06
p38/JNK	FOS	1.51	1.46E-05	7.91E-05
p38/JNK	ZFP36	1.33	5.50E-06	4.09E-05
p38/JNK	ETS1	1.29	2.62E-05	1.25E-04
ROS	SOD2	1.19	5.01E-06	3.97E-05
ROS	HMOX1	1.35	4.54E-04	1.38E-03
UPR/ER stress	ATF3	2.76	2.55E-12	3.04E-10
UPR/ER Stress	PPP1R15A	2.17	9.91E-10	3.68E-08
UPR/ER Stress	PLA2G6	1.58	3.80E-07	5.65E-06
UPR/ER Stress	HERPUD1	1.08	1.39E-04	5.01E-04

floating, atmospheric oxygen vs in vivo

process	ID	logFC	P.Value	adj.P.Val
apoptosis	HRK	2.76	1.85E-07	2.76E-06
apoptosis	TNFRSF10D	1.96	1.58E-09	5.92E-08
apoptosis	BNIP3	1.54	2.45E-07	3.24E-06
apoptosis	IER3	1.48	1.99E-06	1.69E-05
apoptosis	ALOX12	1.44	7.20E-06	4.29E-05
apoptosis	TNFRSF10B	1.38	3.33E-07	3.96E-06
apoptosis	BCL2L11	1.31	3.40E-06	2.40E-05
cell cycle	IGFBP3	2.30	1.34E-10	7.98E-09
cell cycle	CDKN1B	1.68	1.67E-07	2.76E-06
cell cycle	MET	1.08	9.12E-06	5.17E-05
cell cycle	EGFR	1.04	1.26E-05	6.83E-05
cell cycle	TP63	-1.22	9.85E-05	4.34E-04
DDR	MSH2	-1.02	3.01E-06	2.39E-05
DDR	RAD51	-1.09	2.80E-03	7.58E-03
DDR	SESN1	-1.11	7.23E-05	3.31E-04
DDR	BLM	-1.31	1.25E-06	1.24E-05
DDR	BRCA1	-1.38	9.00E-08	1.79E-06
DDR	FANCM	-1.61	2.68E-08	6.39E-07
necrosis/autoph.	PVR	1.33	3.87E-06	2.56E-05
p38/JNK	MAFF	1.73	7.91E-07	8.56E-06
p38/JNK	FOS	1.44	2.59E-05	1.28E-04
ROS	HMOX1	1.15	1.78E-03	5.16E-03
UPR/ER stress	ATF3	3.01	6.04E-13	7.19E-11
UPR/ER Stress	PPP1R15A	2.08	1.99E-09	5.92E-08
UPR/ER Stress	HERPUD1	1.57	1.62E-06	1.48E-05
UPR/ER Stress	HSPA5	1.35	3.43E-06	2.40E-05
UPR/ER Stress	PLA2G6	1.28	6.01E-06	3.76E-05

floating, low oxygen vs in vivo

process	ID	logFC	P.Value	adj.P.Val
apoptosis	HRK	2.97	6.54E-08	5.56E-07
apoptosis	TNFRSF10D	2.47	4.19E-11	2.36E-09
apoptosis	TNFRSF10B	1.63	3.01E-08	3.58E-07
apoptosis	BNIP3	1.43	7.02E-07	4.40E-06
apoptosis	BCL2L11	1.35	2.27E-06	1.08E-05
apoptosis	IER3	1.23	1.91E-05	6.68E-05
apoptosis	CFLAR	-1.04	1.07E-05	3.99E-05
cell cycle	IGFBP3	2.21	2.53E-10	5.03E-09
cell cycle	CDKN1B	1.68	1.70E-07	1.27E-06
cell cycle	EGFR	1.09	7.09E-06	2.91E-05
cell cycle	CDK1	-1.17	3.19E-06	1.41E-05
cell cycle	CHEK1	-1.23	5.83E-07	3.85E-06
cell cycle	CCNB1	-1.29	2.09E-06	1.04E-05
cell cycle	TP63	-3.08	3.44E-10	5.85E-09
DDR	ATR	-1.02	8.73E-06	3.35E-05
DDR	XRCC3	-1.07	7.97E-06	3.16E-05
DDR	SESN1	-1.19	3.31E-05	1.07E-04
DDR	MSH2	-1.33	8.46E-08	6.71E-07
DDR	FANCD2	-1.51	4.29E-08	4.64E-07
DDR	RAD51	-1.61	1.87E-05	6.68E-05
DDR	BRCA1	-2.19	7.93E-11	2.36E-09
DDR	BLM	-2.23	5.26E-10	7.83E-09
DDR	FANCM	-2.26	1.38E-10	3.27E-09
necrosis/autoph.	PVR	1.16	2.06E-05	6.81E-05
p38/JNK	MAFF	1.73	8.04E-07	4.55E-06
p38/JNK	FOS	1.29	8.29E-05	2.47E-04
p38/JNK	EGR1	-1.43	1.89E-07	1.32E-06
p38/JNK	OAS1	-1.76	7.55E-07	4.50E-06
ROS	HMOX1	2.17	2.10E-06	1.04E-05
ROS	HIF1A	-1.27	1.81E-06	9.78E-06
UPR/ER stress	ATF3	3.27	1.51E-13	1.80E-11
UPR/ER Stress	PPP1R15A	2.55	7.75E-11	2.36E-09
UPR/ER Stress	HERPUD1	2.00	5.90E-08	5.48E-07
UPR/ER Stress	HSPA5	1.98	1.71E-08	2.26E-07
UPR/ER Stress	PLA2G6	1.80	5.99E-08	5.48E-07
UPR/ER Stress	EPHB1	-2.18	2.52E-06	1.15E-05

Red indicates significantly up-regulated genes, and green indicates down-regulated genes ($p < 0.05$).
The different stress pathways are indicated by different colors.

Supplementary Table 2: **Part 3: The fold-changes in transcriptional biomarkers observed in slices from PC295 prostate PDX after cultivation.**

PC295 prostate PDX

filter, atmospheric oxygen vs in vivo

process	ID	logFC	P.Value	adj.P.Val
apoptosis	BCL2L14	2.02	5.07E-06	8.33E-05
apoptosis	PMAIP1	1.30	3.58E-06	8.22E-05
apoptosis	BNIP3	1.00	2.23E-03	1.02E-02
cell cycle	CDKN1B	1.39	3.36E-04	2.97E-03
cell cycle	MYC	-1.16	8.20E-04	4.49E-03
cell cycle	TP53	-1.95	2.08E-06	5.98E-05
DDR	XPC	-1.07	6.12E-04	4.15E-03
p38/JNK	MAFF	4.23	7.93E-07	3.04E-05
p38/JNK	EGR1	4.11	1.76E-07	1.01E-05
p38/JNK	FOS	3.69	7.28E-04	4.19E-03
p38/JNK	OAS1	1.28	1.41E-02	4.52E-02
p38/JNK	PHLDA1	1.11	1.41E-02	4.52E-02
p38/JNK	KLF6	1.02	1.26E-04	1.32E-03
ROS	NQO1	1.73	4.55E-06	8.33E-05
ROS	HIF1A	1.03	6.35E-06	9.12E-05
UPR/ER stress	ATF3	4.22	1.15E-09	1.33E-07

filter, low oxygen vs in vivo

process	ID	logFC	P.Value	adj.P.Val
apoptosis	PMAIP1	2.14	1.42E-08	4.09E-07
apoptosis	BNIP3	1.25	3.95E-04	1.30E-03
apoptosis	ZMAT3	-1.02	5.32E-04	1.61E-03
apoptosis	BIK	-1.03	1.46E-03	3.83E-03
apoptosis	TRAF1	-1.25	1.21E-02	2.24E-02
apoptosis	SHISA5	-2.08	1.03E-02	1.97E-02
cell cycle	CDKN1B	1.33	4.67E-04	1.45E-03
cell cycle	BTG2	-1.17	3.92E-03	9.38E-03
cell cycle	PCNA	-1.61	8.25E-07	8.63E-06
cell cycle	TP53	-2.17	6.63E-07	7.62E-06
cell cycle	TERT	-2.45	1.56E-02	2.80E-02
cell cycle	MYC	-2.97	7.53E-08	1.61E-06
DDR	FANCM	-1.24	6.02E-06	3.84E-05
DDR	XRCC1	-1.25	1.02E-03	3.01E-03
DDR	MLH1	-1.34	1.19E-07	1.95E-06
DDR	DDB2	-1.36	1.75E-05	9.61E-05
DDR	RAD51	-1.41	2.72E-06	2.09E-05
DDR	NLRP2	-1.41	8.39E-08	1.61E-06
DDR	XRCC3	-1.43	2.80E-02	4.67E-02
DDR	MSH2	-1.44	4.94E-07	6.31E-06
DDR	ATM	-1.54	1.10E-05	6.34E-05
DDR	FEN1	-1.58	6.25E-05	2.99E-04
DDR	BLM	-1.66	4.37E-06	3.14E-05
DDR	BRCA1	-1.69	2.14E-06	1.84E-05
DDR	FANCG	-1.76	1.15E-03	3.16E-03
DDR	XPC	-1.94	2.24E-06	1.84E-05
DDR	ATR	-2.02	5.97E-09	3.43E-07
necrosis/autoph.	ATG7	-1.07	9.78E-05	4.02E-04
p38/JNK	EGR1	5.24	1.09E-08	4.09E-07
p38/JNK	MAFF	4.50	4.17E-07	5.99E-06
p38/JNK	FOS	4.46	1.48E-04	5.68E-04
p38/JNK	ZFP36	2.71	1.82E-03	4.55E-03
p38/JNK	KLF6	1.62	1.33E-06	1.28E-05
p38/JNK	OAS1	1.49	6.37E-03	1.36E-02
p38/JNK	PHLDA1	1.06	1.83E-02	3.24E-02
ROS	NQO1	1.30	7.90E-05	3.53E-04
UPR/ER stress	ATF3	5.14	1.29E-10	1.48E-08
UPR/ER Stress	HERPUD1	1.59	5.03E-06	3.41E-05
UPR/ER Stress	ATF4	1.46	8.91E-06	5.39E-05
UPR/ER Stress	HSPA5	1.05	1.72E-04	6.37E-04
UPR/ER Stress	PLA2G6	-1.34	6.02E-03	1.31E-02

floating, atmospheric oxygen vs in vivo

process	ID	logFC	P.Value	adj.P.Val
apoptosis	PMAIP1	2.33	5.35E-09	1.54E-07
apoptosis	BNIP3	1.37	1.85E-04	6.46E-04
apoptosis	MCL1	1.13	5.15E-03	1.04E-02
apoptosis	AIFM2	1.02	2.86E-03	6.86E-03
cell cycle	CDKN1B	1.82	2.91E-05	1.34E-04
cell cycle	EGFR	1.50	1.14E-05	7.27E-05
cell cycle	CDKN1A	1.27	1.64E-03	4.23E-03
cell cycle	CHEK1	-1.01	8.92E-04	2.44E-03
cell cycle	PCNA	-1.14	2.77E-05	1.33E-04
cell cycle	TP53	-1.60	1.58E-05	9.56E-05
cell cycle	MYC	-2.33	1.12E-06	1.17E-05
DDR	MLH1	-1.08	1.28E-06	1.23E-05
DDR	FANCG	-1.24	1.20E-02	2.33E-02
DDR	FEN1	-1.35	2.66E-04	8.48E-04
DDR	NLRP2	-1.36	1.28E-07	2.46E-06
DDR	ATM	-1.37	1.75E-04	1.50E-04
DDR	XPC	-1.40	5.90E-05	2.39E-04
DDR	RAD51	-1.41	2.78E-06	2.28E-05
DDR	FANCM	-1.52	7.09E-07	8.16E-06
DDR	MSH2	-1.53	2.55E-07	4.19E-06
DDR	BRCA1	-1.56	4.89E-06	3.51E-05
DDR	BLM	-1.65	4.80E-06	3.51E-05
DDR	ATR	-2.20	2.24E-09	8.58E-08
necrosis/autophagy	PARP1	-1.11	2.75E-05	1.33E-04
p38/JNK	EGR1	6.61	7.23E-10	4.16E-08
p38/JNK	MAFF	6.29	1.21E-08	2.78E-07
p38/JNK	FOS	5.46	2.22E-05	1.16E-04
p38/JNK	ZFP36	4.58	2.02E-05	1.10E-04
p38/JNK	JUN	2.62	2.67E-03	6.67E-03
p38/JNK	OAS1	2.49	2.08E-04	7.04E-04
p38/JNK	PHLDA1	2.25	8.13E-05	3.12E-04
p38/JNK	KLF6	1.81	3.94E-07	5.67E-06
ROS	HMOX1	4.01	3.02E-03	6.93E-03
ROS	NQO1	1.88	1.93E-06	1.71E-05
UPR/ER stress	ATF3	5.89	2.87E-11	3.30E-09
UPR/ER Stress	PPP1R15A	5.29	1.12E-04	4.16E-04
UPR/ER Stress	HERPUD1	1.98	4.90E-07	6.26E-06
UPR/ER Stress	HSPA5	1.40	1.07E-05	7.24E-05
UPR/ER Stress	ATF4	1.20	6.02E-05	2.39E-04

floating, low oxygen vs in vivo

process	ID	logFC	P.Value	adj.P.Val
apoptosis	PMAIP1	2.99	2.80E-10	1.61E-08
apoptosis	TNFRSF10B	1.22	1.52E-04	4.61E-04
apoptosis	MCL1	1.21	3.36E-03	6.43E-03
apoptosis	BNIP3	1.00	2.19E-03	4.50E-03
apoptosis	ZMAT3	-1.10	2.75E-04	7.72E-04
apoptosis	TNFRSF10A	-1.31	7.15E-05	2.42E-04
apoptosis	PIDD	-1.58	1.06E-02	1.84E-02
cell cycle	CDKN1B	1.93	1.59E-05	6.31E-05
cell cycle	CDKN1A	1.14	3.49E-03	6.58E-03
cell cycle	TP73	-1.09	2.73E-02	4.19E-02
cell cycle	CCNA2	-1.10	1.77E-03	3.83E-03
cell cycle	CDC25C	-1.12	3.53E-04	9.68E-04
cell cycle	RPA1	-1.13	1.26E-05	5.54E-05
cell cycle	CDK1	-1.25	3.93E-06	2.15E-05
cell cycle	BTG2	-1.26	2.39E-03	4.82E-03
cell cycle	TP53	-1.78	5.57E-06	2.67E-05
cell cycle	PCNA	-1.87	1.60E-07	1.53E-06
cell cycle	TERT	-2.60	1.59E-02	2.65E-02
cell cycle	MYC	-2.99	6.88E-08	7.92E-07
DDR	XRCC1	-1.00	4.78E-03	8.60E-03
DDR	DDB2	-1.21	5.58E-05	1.94E-04
DDR	ATM	-1.26	7.61E-05	2.50E-04
DDR	SESN1	-1.29	4.54E-06	2.27E-05
DDR	MLH1	-1.43	5.68E-08	7.54E-07
DDR	XRCC3	-1.44	2.69E-02	4.18E-02
DDR	RAD51	-1.45	2.09E-06	1.34E-05
DDR	BLM	-1.49	1.39E-05	5.70E-05
DDR	FANCM	-1.54	6.12E-07	5.41E-06
DDR	BRCA1	-1.65	2.83E-06	1.63E-05
DDR	MSH2	-1.67	9.37E-08	9.80E-07
DDR	FANCG	-1.79	1.04E-03	2.43E-03
DDR	NLRP2	-1.98	1.69E-09	3.88E-08
DDR	FEN1	-1.99	6.66E-06	3.06E-05
DDR	XPC	-2.02	1.45E-06	1.04E-05
DDR	ATR	-2.43	7.01E-10	2.01E-08
necrosis/autoph.	PARP1	-1.52	1.13E-06	8.68E-06
necrosis/autoph.	RIPK1	-1.97	2.75E-06	1.63E-05
p38/JNK	EGR1	6.89	4.35E-10	1.67E-08
p38/JNK	MAFF	6.47	8.93E-09	1.47E-07
p38/JNK	FOS	5.56	1.85E-05	6.84E-05
p38/JNK	ZFP36	4.78	1.30E-05	5.54E-05
p38/JNK	OAS1	3.23	8.06E-05	2.50E-04
p38/JNK	PHLDA1	2.62	1.90E-05	6.84E-05
p38/JNK	KLF6	2.14	5.90E-08	7.54E-07
p38/JNK	JUN	2.02	1.32E-02	2.27E-02
ROS	HMOX1	3.36	9.14E-03	1.62E-02
UPR/ER stress	ATF3	6.38	1.16E-11	1.33E-09
UPR/ER Stress	PPP1R15A	4.18	8.25E-04	1.98E-03
UPR/ER Stress	HERPUD1	2.86	7.72E-09	1.47E-07
UPR/ER Stress	HSPA5	1.77	9.57E-07	7.86E-06
UPR/ER Stress	ATF4	1.57	4.16E-06	2.18E-05
UPR/ER Stress	XBP1(U)	-1.04	2.16E-04	6.38E-04
UPR/ER Stress	EDEM2	-1.07	1.73E-05	6.63E-05

Red indicates significantly up-regulated genes, and green indicates down-regulated genes ($p < 0.05$).
The different stress pathways are indicated by different colors.

Supplementary Table 2: **Part 4: The fold-changes in transcriptional biomarkers observed in slices from MCF-7 breast CDX after cultivation.**

MCF-7 breast CDX

filter, atmospheric oxygen vs in vivo

process	ID	logFC	P.Value	adj.P.Val
apoptosis	HRK	1.31	2.78E-03	8.42E-03
apoptosis	BID	-1.02	3.72E-03	1.07E-02
apoptosis	BCL2L11	-1.17	6.78E-05	4.33E-04
apoptosis	PIDD	-1.99	4.15E-03	1.14E-02
apoptosis	TP53AIP1	-2.10	6.97E-06	8.02E-05
cell cycle	CCNB1	-1.03	1.22E-04	7.00E-04
cell cycle	CHEK1	-1.03	1.49E-05	1.32E-04
cell cycle	BTG2	-1.08	1.24E-02	2.91E-02
cell cycle	CDC25C	-1.22	2.41E-07	4.61E-06
cell cycle	CCNA2	-1.32	2.74E-05	1.97E-04
cell cycle	TERT	-1.35	8.48E-03	2.17E-02
cell cycle	SFN	-1.95	7.43E-05	4.50E-04
cell cycle	TP63	-2.81	1.84E-08	1.77E-06
cell cycle	TAp73	-2.89	5.49E-05	3.71E-04
cell cycle	TP73	-3.28	2.81E-07	4.61E-06
DDR	ATR	1.07	7.60E-04	2.91E-03
DDR	XPA	-1.05	2.11E-04	1.16E-03
DDR	BRCA1	-1.19	8.44E-08	2.43E-06
DDR	BLM	-1.47	1.88E-07	4.32E-06
DDR	DDB2	-1.49	4.61E-08	1.77E-06
DDR	RAD51	-1.76	3.18E-04	1.52E-03
p38/JNK	OAS1	-1.06	4.07E-03	1.14E-02
p38/JNK	EGR1	-2.28	9.28E-06	8.89E-05
ROS	HMOX1	1.77	9.44E-07	1.36E-05
ROS	SQSTM1	1.02	6.85E-04	2.81E-03
ROS	SOD2	-1.15	4.34E-06	5.55E-05
UPR/ER Stress	HERPUD1	2.08	3.40E-08	1.77E-06
UPR/ER Stress	HSPA5	1.40	2.61E-05	1.97E-04

filter, low oxygen vs in vivo

process	ID	logFC	P.Value	adj.P.Val
apoptosis	TP53AIP1	-1.36	9.84E-04	4.92E-03
cell cycle	CCNB1	-1.20	2.00E-05	1.92E-04
cell cycle	CCNA2	-1.37	1.84E-05	1.92E-04
cell cycle	TERT	-1.41	6.32E-03	2.20E-02
cell cycle	SFN	-1.51	1.07E-03	5.12E-03
cell cycle	CDC25C	-1.56	3.93E-09	4.52E-07
cell cycle	TAp73	-1.73	7.20E-03	2.44E-02
cell cycle	TP73	-1.91	3.35E-04	1.92E-03
cell cycle	TP63	-2.10	1.89E-06	2.72E-05
DDR	BRCA1	-1.03	8.42E-07	1.61E-05
DDR	BLM	-1.27	1.76E-06	2.72E-05
DDR	RAD51	-1.44	2.00E-05	1.92E-04
DDR	DDB2	-1.54	2.74E-08	7.88E-07
p38/JNK	EGR1	-2.10	2.69E-05	2.38E-04
ROS	HIF1A	1.18	1.97E-03	8.40E-03
ROS	SOD1	-1.02	5.06E-05	3.64E-04
ROS	SOD2	-1.64	1.59E-08	7.88E-07
UPR/ER Stress	HERPUD1	2.12	2.52E-08	7.88E-07
UPR/ER Stress	HSPA5	1.58	4.83E-06	6.17E-05
UPR/ER Stress	DNAJC3	1.35	4.38E-08	1.01E-06

Red indicates significantly up-regulated genes, and green indicates down-regulated genes ($p < 0.05$).
The different stress pathways are indicated by different colors.

References:

1. Johnson, R. A. Transcriptional Repression by p53 through Direct Binding to a Novel DNA Element. *J. Biol. Chem.* **276,** 27716–27720 (2001).

2. Wu, M. *et al.* AMID is a p53-inducible gene downregulated in tumors. *Oncogene* **23,** 6815–6819 (2004).

3. Arakawa, T., Nakamura, M., Yoshimoto, T. & Yamamoto, S. The transcriptional regulation of human arachidonate 12-lipoxygenase gene by NF kappa B/Rel. *FEBS Lett.* **363,** 105–110 (1995).

4. Robles, A. I., Bemmels, N. A., Foraker, A. B. & Harris, C. C. APAF-1 is a transcriptional target of p53 in DNA damage-induced apoptosis. *Cancer Res.* **61,** 6660–6664 (2001).

5. Lv, G. *et al.* MicroRNA-451 regulates activating transcription factor 2 expression and inhibits liver cancer cell migration. *Oncol. Rep.* (2014). doi:10.3892/or.2014.3296

6. Zhang, C. *et al.* Transcriptional activation of the human stress-inducible transcriptional repressor ATF3 gene promoter by p53. *Biochem. Biophys. Res. Commun.* **297,** 1302–1310 (2002).

7. Kim, I., Xu, W. & Reed, J. C. Cell death and endoplasmic reticulum stress: disease relevance and therapeutic opportunities. *Nat. Rev. Drug Discov.* **7,** 1013–1030 (2008).

8. Adachi, Y. *et al.* ATF6 Is a Transcription Factor Specializing in the Regulation of Quality Control Proteins in the Endoplasmic Reticulum. *Cell Struct. Funct.* **33,** 75–89 (2008).

9. Verfaillie, T., Salazar, M., Velasco, G. & Agostinis, P. Linking ER Stress to Autophagy: Potential Implications for Cancer Therapy. *Int. J. Cell Biol.* **2010,** e930509 (2010).

10. Maskey, D. *et al.* ATG5 is induced by DNA-damaging agents and promotes mitotic catastrophe independent of autophagy. *Nat. Commun.* **4,** (2013).

11. Comincini, S. *et al.* microRNA-17 regulates the expression of ATG7 and modulates the autophagy process, improving the sensitivity to temozolomide and low-dose ionizing radiation treatments in human glioblastoma cells. *Cancer Biol. Ther.* **14,** 574–586 (2013).

12. Moiola, C. *et al.* Dynamic coregulatory complex containing BRCA1, E2F1 and CtIP controls ATM transcription. *Cell. Physiol. Biochem. Int. J. Exp. Cell. Physiol. Biochem. Pharmacol.* **30,** 596–608 (2012).

13. Wang, X. *et al.* HOXC9 directly regulates distinct sets of genes to coordinate diverse cellular processes during neuronal differentiation. *BMC Genomics* **14,** 830 (2013).

14. Graupner, V. *et al.* Differential regulation of the proapoptotic multidomain protein Bak by p53 and p73 at the promoter level. *Cell Death Differ.* **18,** 1130–1139 (2011).

15. Catz, S. D. & Johnson, J. L. Transcriptional regulation of bcl-2 by nuclear factor kappa B and its significance in prostate cancer. *Oncogene* **20,** 7342–7351 (2001).

16. Riley, T., Sontag, E., Chen, P. & Levine, A. Transcriptional control of human p53-regulated genes. *Nat. Rev. Mol. Cell Biol.* **9,** 402–412 (2008).

17. Wei, C.-L. *et al.* A global map of p53 transcription-factor binding sites in the human genome. *Cell* **124,** 207–219 (2006).

18. Grumont, R. J., Rourke, I. J. & Gerondakis, S. Rel-dependent induction of A1 transcription is required to protect B cells from antigen receptor ligation-induced apoptosis. *Genes Dev.* **13,** 400–411 (1999).

19. Lee, R. M., Gillet, G., Burnside, J., Thomas, S. J. & Neiman, P. Role of Nr13 in regulation of programmed cell death in the bursa of Fabricius. *Genes Dev.* **13,** 718–728 (1999).

20. Wang, Z., Zhang, B., Yang, L., Ding, J. & Ding, H.-F. Constitutive production of NF-kappaB2 p52 is not tumorigenic but predisposes mice to inflammatory autoimmune disease by repressing Bim expression. *J. Biol. Chem.* **283,** 10698–10706 (2008).

21. Miled, C., Pontoglio, M., Garbay, S., Yaniv, M. & Weitzman, J. B. A genomic map of p53 binding sites identifies novel p53 targets involved in an apoptotic network. *Cancer Res.* **65,** 5096–5104 (2005).

22. Copetti, T., Bertoli, C., Dalla, E., Demarchi, F. & Schneider, C. p65/RelA modulates BECN1 transcription and autophagy. *Mol. Cell. Biol.* **29,** 2594–2608 (2009).

23. Sax, J. K. *et al.* BID regulation by p53 contributes to chemosensitivity. *Nat. Cell Biol.* **4,** 842–849 (2002).

24. Chinnadurai, G., Vijayalingam, S. & Rashmi, R. BIK, the founding member of the BH3-only family proteins: mechanisms of cell death and role in cancer and pathogenic processes. *Oncogene* **27 Suppl 1,** S20–29 (2008).

25. Stehlik, C. *et al.* Nuclear factor (NF)-kappaB-regulated X-chromosome-linked iap gene expression protects endothelial cells from tumor necrosis factor alpha-induced apoptosis. *J. Exp. Med.* **188,** 211–216 (1998).

26. Slupianek, A. *et al.* BLM helicase is activated in BCR/ABL leukemia cells to modulate responses to cisplatin. *Oncogene* **24,** 3914–3922 (2005).

27. Baetz, D. *et al.* Nuclear factor-kappaB-mediated cell survival involves transcriptional silencing of the mitochondrial death gene BNIP3 in ventricular myocytes. *Circulation* **112,** 3777–3785 (2005).

28. Yakovlev, A. G. *et al.* BOK and NOXA are essential mediators of p53-dependent apoptosis. *J. Biol. Chem.* **279,** 28367–28374 (2004).

29. Thakur, S. *et al.* Regulation of BRCA1 Transcription by Specific Single-Stranded DNA Binding Factors. *Mol. Cell. Biol.* **23,** 3774–3787 (2003).

30. Rivera, A., Mavila, A., Bayless, K. J., Davis, G. E. & Maxwell, S. A. Cyclin A1 is a p53-induced gene that mediates apoptosis, G2/M arrest, and mitotic catastrophe in renal, ovarian, and lung carcinoma cells. *Cell. Mol. Life Sci. CMLS* **63,** 1425–1439 (2006).

31. Innocente, S. A., Abrahamson, J. L., Cogswell, J. P. & Lee, J. M. p53 regulates a G2 checkpoint through cyclin B1. *Proc. Natl. Acad. Sci. U. S. A.* **96,** 2147–2152 (1999).

32. Okamoto, K. & Beach, D. Cyclin G is a transcriptional target of the p53 tumor suppressor protein. *EMBO J.* **13,** 4816–4822 (1994).

33. Mori, T. *et al.* Cyclin K as a direct transcriptional target of the p53 tumor suppressor. *Neoplasia N. Y. N* **4,** 268–274 (2002).

34. St Clair, S. *et al.* DNA damage-induced downregulation of Cdc25C is mediated by p53 via two independent mechanisms: one involves direct binding to the cdc25C promoter. *Mol. Cell* **16,** 725–736 (2004).

35. Yun, J. *et al.* p53 negatively regulates cdc2 transcription via the CCAAT-binding NF-Y transcription factor. *J. Biol. Chem.* **274,** 29677–29682 (1999).

36. Hinata, K., Gervin, A. M., Jennifer Zhang, Y. & Khavari, P. A. Divergent gene regulation and growth effects by NF-kappa B in epithelial and mesenchymal cells of human skin. *Oncogene* **22,** 1955–1964 (2003).

37. Chu, I. M., Hengst, L. & Slingerland, J. M. The Cdk inhibitor p27 in human cancer: prognostic potential and relevance to anticancer therapy. *Nat. Rev. Cancer* **8,** 253–267 (2008).

38. Kreuz, S., Siegmund, D., Scheurich, P. & Wajant, H. NF-kappaB inducers upregulate cFLIP, a cycloheximide-sensitive inhibitor of death receptor signaling. *Mol. Cell. Biol.* **21,** 3964–3973 (2001).

39. Matsui, T. *et al.* Negative regulation of Chk2 expression by p53 is dependent on the CCAAT-binding transcription factor NF-Y. *J. Biol. Chem.* **279,** 25093–25100 (2004).

40. Samali, A., FitzGerald, U., Deegan, S. & Gupta, S. Methods for Monitoring Endoplasmic Reticulum Stress and the Unfolded Protein Response. *Int. J. Cell Biol.* **2010,** e830307 (2010).

41. Olivari, S., Galli, C., Alanen, H., Ruddock, L. & Molinari, M. A novel stress-induced EDEM variant regulating endoplasmic reticulum-associated glycoprotein degradation. *J. Biol. Chem.* **280,** 2424–2428 (2005).

42. Nishi, H. *et al.* Analysis of the epidermal growth factor receptor promoter: the effect of nuclear factor-kappaB. *Int. J. Mol. Med.* **11,** 49–55 (2003).

43. Zhou, A., Scoggin, S., Gaynor, R. B. & Williams, N. S. Identification of NF-kappa B-regulated genes induced by TNFalpha utilizing expression profiling and RNA interference. *Oncogene* **22,** 2054–2064 (2003).

44. Ferreiro, I. *et al.* Whole genome analysis of p38 SAPK-mediated gene expression upon stress. *BMC Genomics* **11,** 144 (2010).

45. Yan, Q.-W. *et al.* MZF1 possesses a repressively regulatory function in ERCC1 expression. *Biochem. Pharmacol.* **71,** 761–771 (2006).

46. Tanaka, K., Oda, N., Iwasaka, C., Abe, M. & Sato, Y. Induction of Ets-1 in endothelial cells during reendothelialization after denuding injury. *J. Cell. Physiol.* **176,** 235–244 (1998).

47. Li, N., Zhang, F., Li, S. & Zhou, S. Epigenetic silencing of MicroRNA-503 regulates FANCA expression in non-small cell lung cancer cell. *Biochem. Biophys. Res. Commun.* **444,** 611–616 (2014).

48. Scanlon, S. E. & Glazer, P. M. Hypoxic Stress Facilitates Acute Activation and Chronic Downregulation of Fanconi Anemia Proteins. *Mol. Cancer Res. MCR* (2014). doi:10.1158/1541-7786.MCR-13-0628

49. Tsai, Y.-S. *et al.* Areca nut induces miR-23a and inhibits repair of DNA double-strand breaks by targeting FANCG. *Toxicol. Sci. Off. J. Soc. Toxicol.* **123,** 480–490 (2011).

50. Chan, H., Bartos, D. P. & Owen-Schaub, L. B. Activation-dependent transcriptional regulation of the human Fas promoter requires NF-kappaB p50-p65 recruitment. *Mol. Cell. Biol.* **19,** 2098–2108 (1999).

51. Matsui, K., Fine, A., Zhu, B., Marshak-Rothstein, A. & Ju, S. T. Identification of two NF-kappa B sites in mouse CD95 ligand (Fas ligand) promoter: functional analysis in T cell hybridoma. *J. Immunol. Baltim. Md 1950* **161,** 3469–3473 (1998).

52. Christmann, M., Tomicic, M. T., Origer, J. & Kaina, B. Fen1 is induced p53 dependently and involved in the recovery from UV-light-induced replication inhibition. *Oncogene* **24,** 8304–8313 (2005).

53. Kwak, E. L., Larochelle, D. A., Beaumont, C., Torti, S. V. & Torti, F. M. Role for NF-kappa B in the regulation of ferritin H by tumor necrosis factor-alpha. *J. Biol. Chem.* **270,** 15285–15293 (1995).

54. Xia, C., Hu, J., Ketterer, B. & Taylor, J. B. The organization of the human GSTP1-1 gene promoter and its response to retinoic acid and cellular redox status. *Biochem. J.* **313 (Pt 1),** 155–161 (1996).

55. Harrison, P. M. & Farzaneh, F. Regulation of HGF/SF gene expression in MRC-5 cells by N-acetylcysteine. *Biochem. Biophys. Res. Commun.* **279,** 108–115 (2000).

56. Bonello, S. *et al.* Reactive oxygen species activate the HIF-1alpha promoter via a functional NFkappaB site. *Arterioscler. Thromb. Vasc. Biol.* **27,** 755–761 (2007).

57. Liu, Y., Bernauer, A. M., Yingling, C. M. & Belinsky, S. A. HIF1α regulated expression of XPA contributes to cisplatin resistance in lung cancer. *Carcinogenesis* **33,** 1187–1192 (2012).

58. Lavrovsky, Y., Schwartzman, M. L., Levere, R. D., Kappas, A. & Abraham, N. G. Identification of binding sites for transcription factors NF-kappa B and AP-2 in the promoter region of the human heme oxygenase 1 gene. *Proc. Natl. Acad. Sci. U. S. A.* **91,** 5987–5991 (1994).

59. Imaizumi, K. *et al.* Molecular cloning of a novel polypeptide, DP5, induced during programmed neuronal death. *J. Biol. Chem.* **272,** 18842–18848 (1997).

60. Ammirante, M. *et al.* The activity of hsp90 alpha promoter is regulated by NF-kappa B transcription factors. *Oncogene* **27,** 1175–1178 (2008).

61. Wu, M. X., Ao, Z., Prasad, K. V., Wu, R. & Schlossman, S. F. IEX-1L, an apoptosis inhibitor involved in NF-kappaB-mediated cell survival. *Science* **281,** 998–1001 (1998).

62. Whitmarsh, A. J. A central role for p38 MAPK in the early transcriptional response to stress. *BMC Biol.* **8,** 47 (2010).

63. Tiwari, N. *et al.* Klf4 is a transcriptional regulator of genes critical for EMT, including Jnk1 (Mapk8). *PloS One* **8,** e57329 (2013).

64. Juin, P., Geneste, O., Gautier, F., Depil, S. & Campone, M. Decoding and unlocking the BCL-2 dependency of cancer cells. *Nat. Rev. Cancer* **13,** 455–465 (2013).

65. Duyao, M. P., Buckler, A. J. & Sonenshein, G. E. Interaction of an NF-kappa B-like factor with a site upstream of the c-myc promoter. *Proc. Natl. Acad. Sci. U. S. A.* **87,** 4727–4731 (1990).

66. Fontalba, A., Gutierrez, O. & Fernandez-Luna, J. L. NLRP2, an inhibitor of the NF-kappaB pathway, is transcriptionally activated by NF-kappaB and exhibits a nonfunctional allelic variant. *J. Immunol. Baltim. Md 1950* **179,** 8519–8524 (2007).

67. Geller, D. A. *et al.* Cytokine induction of interferon regulatory factor-1 in hepatocytes. *Surgery* **114,** 235–242 (1993).

68. Yao, K. S. & O'Dwyer, P. J. Involvement of NF-kappa B in the induction of NAD(P)H:quinone oxidoreductase (DT-diaphorase) by hypoxia, oltipraz and mitomycin C. *Biochem. Pharmacol.* **49,** 275–282 (1995).

69. Lin, Y., Ma, W. & Benchimol, S. Pidd, a new death-domain-containing protein, is induced by p53 and promotes apoptosis. *Nat. Genet.* **26,** 122–127 (2000).

70. Acosta-Alvear, D. *et al.* XBP1 controls diverse cell type- and condition-specific transcriptional regulatory networks. *Mol. Cell* **27,** 53–66 (2007).

71. Marciniak, S. J. *et al.* CHOP induces death by promoting protein synthesis and oxidation in the stressed endoplasmic reticulum. *Genes Dev.* **18,** 3066–3077 (2004).

72. Kim, Y.-J. *et al.* Human prx1 gene is a target of Nrf2 and is up-regulated by hypoxia/reoxygenation: implication to tumor biology. *Cancer Res.* **67,** 546–554 (2007).

73. Ardolino, M. *et al.* DNAM-1 ligand expression on Ag-stimulated T lymphocytes is mediated by ROS-dependent activation of DNA-damage response: relevance for NK-T cell interaction. *Blood* **117,** 4778–4786 (2011).

74. Nakano, K., Bálint, E., Ashcroft, M. & Vousden, K. H. A ribonucleotide reductase gene is a transcriptional target of p53 and p73. *Oncogene* **19,** 4283–4289 (2000).

75. Lee, M. H., Mabb, A. M., Gill, G. B., Yeh, E. T. H. & Miyamoto, S. NF-κB induction of the SUMO protease SENP2: A negative feedback loop to attenuate cell survival response to genotoxic stress. *Mol. Cell* **43,** 180–191 (2011).

76. Hermeking, H. *et al.* 14-3-3 sigma is a p53-regulated inhibitor of G2/M progression. *Mol. Cell* **1,** 3–11 (1997).

77. Bourdon, J.-C., Renzing, J., Robertson, P. L., Fernandes, K. N. & Lane, D. P. Scotin, a novel p53-inducible proapoptotic protein located in the ER and the nuclear membrane. *J. Cell Biol.* **158,** 235–246 (2002).

78. Milner, J. Cellular regulation of SIRT1. *Curr. Pharm. Des.* **15,** 39–44 (2009).

79. Rojo, A. I., Salinas, M., Martín, D., Perona, R. & Cuadrado, A. Regulation of Cu/Zn-superoxide dismutase expression via the phosphatidylinositol 3 kinase/Akt pathway and nuclear factor-kappaB. *J. Neurosci. Off. J. Soc. Neurosci.* **24,** 7324–7334 (2004).

80. Xu, Y. *et al.* Mutations in the promoter reveal a cause for the reduced expression of the human manganese superoxide dismutase gene in cancer cells. *Oncogene* **18,** 93–102 (1999).

81. Jain, A. *et al.* p62/SQSTM1 is a target gene for transcription factor NRF2 and creates a positive feedback loop by inducing antioxidant response element-driven gene transcription. *J. Biol. Chem.* **285,** 22576–22591 (2010).

82. Chen, X., Zheng, Y., Zhu, J., Jiang, J. & Wang, J. p73 is transcriptionally regulated by DNA damage, p53, and p73. *Oncogene* **20,** 769–774 (2001).

83. Yin, L., Hubbard, A. K. & Giardina, C. NF-kappa B regulates transcription of the mouse telomerase catalytic subunit. *J. Biol. Chem.* **275,** 36671–36675 (2000).

84. Liu, X., Yue, P., Khuri, F. R. & Sun, S.-Y. p53 upregulates death receptor 4 expression through an intronic p53 binding site. *Cancer Res.* **64,** 5078–5083 (2004).

85. Liu, X., Yue, P., Khuri, F. R. & Sun, S.-Y. Decoy receptor 2 (DcR2) is a p53 target gene and regulates chemosensitivity. *Cancer Res.* **65,** 9169–9175 (2005).

86. Wu, H. & Lozano, G. NF-kappa B activation of p53. A potential mechanism for suppressing cell growth in response to stress. *J. Biol. Chem.* **269,** 20067–20074 (1994).

87. O'Prey, J. *et al.* p53-mediated induction of Noxa and p53AIP1 requires NFkappaB. *Cell Cycle Georget. Tex* **9,** 947–952 (2010).

88. Schwenzer, R. *et al.* The human tumor necrosis factor (TNF) receptor-associated factor 1 gene (TRAF1) is up-regulated by cytokines of the TNF ligand family and modulates TNF-induced activation of NF-kappaB and c-Jun N-terminal kinase. *J. Biol. Chem.* **274,** 19368–19374 (1999).

89. Wang, C. Y., Mayo, M. W., Korneluk, R. G., Goeddel, D. V. & Baldwin, A. S. NF-kappaB antiapoptosis: induction of TRAF1 and TRAF2 and c-IAP1 and c-IAP2 to suppress caspase-8 activation. *Science* **281,** 1680–1683 (1998).

90. Hawkes, H.-J. K., Karlenius, T. C. & Tonissen, K. F. Regulation of the human thioredoxin gene promoter and its key substrates: a study of functional and putative regulatory elements. *Biochim. Biophys. Acta* **1840,** 303–314 (2014).

91. Byrd, A. E. & Brewer, J. W. Intricately Regulated: A Cellular Toolbox for Fine-Tuning XBP1 Expression and Activity. *Cells* **1,** 738–753 (2012).

92. Turner, D. J. *et al.* Bile salts induce resistance to apoptosis through NF-kappaB-mediated XIAP expression. *Ann. Surg.* **245,** 415–425 (2007).

93. Chen, D., Yu, Z., Zhu, Z. & Lopez, C. D. E2F1 regulates the base excision repair gene XRCC1 and promotes DNA repair. *J. Biol. Chem.* **283,** 15381–15389 (2008).

94. Yanagisawa, T., Urade, M., Yamamoto, Y. & Furuyama, J. Increased expression of human DNA repair genes, XRCC1, XRCC3 and RAD51, in radioresistant human KB carcinoma cell line N10. *Oral Oncol.* **34,** 524–528 (1998).

95. Hellborg, F. *et al.* Human wig-1, a p53 target gene that encodes a growth inhibitory zinc finger protein. *Oncogene* **20,** 5466–5474 (2001).
